# Supplementary material for: Characterization of the Ergosterol Biosynthesis Pathway in Ceratocystidaceae
Source: J Fungi (Basel). 2021 Mar 22;7(3):237. doi: 10.3390/jof7030237 (PMC8004197; doi:10.3390/jof7030237)
Supplement: Supplementary file 1 [file jof-07-00237-s001.zip › Supplementary file S-1.docx]

**Supplementary file S1**

***(Sayari et al – Ceratocystidaceae* terpene biosynthesis gene clusters*)***

The tables below show the sizes and positions of genes in the respective terpene clusters within the relevant contigs, together with the blast scores for the top database hits (i.e, E value, % Coverage, % identity and the Accession number of the top hit).

| B. fagacearum (A) - Contig 380 | | | | | | | |
| --- | --- | --- | --- | --- | --- | --- | --- |
| Predicted gene function | Position on contig | Size (aa) | Closest Species Ortholog | E-value | % Coverage | % Identity | NCBI Accession Number |
| Squalene synthase | 2142-3978 (+) | 467 | [*Neurospora crassa*](https://blast.ncbi.nlm.nih.gov/Blast.cgi#alnHdr_759000359) | 0.0 | 98 | 63 | XP_011395224 |
| Alkaline protease 1 | 5012-6253 (-) | 233 | [*Scedosporium apiospermum*](https://blast.ncbi.nlm.nih.gov/Blast.cgi#alnHdr_1027069126) | 4e-119 | 96 | 78 | XP_016641262 |
| Vacuolar protein sorting-associated protein | 9910-11202 (-) | 347 | [*Neonectria ditissima*](https://blast.ncbi.nlm.nih.gov/Blast.cgi#alnHdr_936406736) | 0.0 | 98 | 82 | KPM35748 |

| B. fagacearum (B) - Contig 31 | | | | | | | |
| --- | --- | --- | --- | --- | --- | --- | --- |
| Predicted gene function | Position on contig | Size (aa) | Closest Species Ortholog | E-value | % Coverage | % Identity | NCBI Accession Number |
| [Dual specificity protein kinase pom1](https://blast.ncbi.nlm.nih.gov/Blast.cgi#alnHdr_1267069088) | 7471-11820 (-) | *1449* | [*Colletotrichum graminicola*](https://blast.ncbi.nlm.nih.gov/Blast.cgi#alnHdr_827061431) | 0.0 | 99 | 55 | XP_008092293 |
| Peroxisomal membrane protein PEX29 | 18271-19626 (+) | 451 | [*Trichoderma reesei*](https://blast.ncbi.nlm.nih.gov/Blast.cgi#alnHdr_589112901) | 0.0 | 90 | 62 | XP_006968473 |
| [Geranylgeranyl pyrophosphate synthase](https://blast.ncbi.nlm.nih.gov/Blast.cgi#alnHdr_1267069085) | 20100-21710 (-) | 536 | [*Lomentospora prolificans*](https://blast.ncbi.nlm.nih.gov/Blast.cgi#alnHdr_1315198120) | 0.0 | 80 | 67 | PKS07637 |
| Hypothetical | 24028-25770 (-) | 580 | [*Verticillium longisporum*](https://blast.ncbi.nlm.nih.gov/Blast.cgi#alnHdr_913903447) | 2e-125 | 84 | 50 | CRJ99998 |
| Hypothetical | 29883-37122 (-) | 1384 | *Chaetomium thermophilum* | 2e-142 | 61 | 36 | XP_006695049 |

| C. adiposa (A) - Contig 34 | | | | | | | |
| --- | --- | --- | --- | --- | --- | --- | --- |
| Predicted gene function | Position on contig | Size (aa) | Closest Species Ortholog | E-value | % Coverage | % Identity | NCBI Accession Number |
| [Glycylpeptide N-tetradecanoyltransferase](https://blast.ncbi.nlm.nih.gov/Blast.cgi#alnHdr_814598560) |  | 92 | [*Sporothrix insectorum*](https://blast.ncbi.nlm.nih.gov/Blast.cgi#alnHdr_1025673247) | 8e-39 | 94 | 76 | OAA60584 |
| Hypothetical | 931-6582 (+) | 331 | [*Verticillium longisporum*](https://blast.ncbi.nlm.nih.gov/Blast.cgi#alnHdr_913803165) | 2e-95 | 100 | 52 | CRK09967 |
| [DNAJ domain containing protein](https://blast.ncbi.nlm.nih.gov/Blast.cgi#alnHdr_531862113) | 16106-17830 (+) | 517 | [*Ophiocordyceps camponoti-rufipedis*](https://blast.ncbi.nlm.nih.gov/Blast.cgi#alnHdr_1267103008) | 0.0 | 98 | 61 | PHH72218 |
| [squalene synthase](https://blast.ncbi.nlm.nih.gov/Blast.cgi#alnHdr_1267071811) | 19967-21917 (+) | 464 | [*Thielavia terrestris*](https://blast.ncbi.nlm.nih.gov/Blast.cgi#alnHdr_367047549) | 0.0 | 93 | 67 | XP_003654154 |
| [Alkaline protease 1](https://blast.ncbi.nlm.nih.gov/Blast.cgi#alnHdr_814598565) | 22877-24365 (-) | 442 | [*Scedosporium apiospermum*](https://blast.ncbi.nlm.nih.gov/Blast.cgi#alnHdr_1027069126) | 0.0 | 89 | 67 | XP_016641262 |
| [Delta (24)-sterol reductase](https://blast.ncbi.nlm.nih.gov/Blast.cgi#alnHdr_1267071833) | 29846-31348 (-) | 500 | [*Fonsecaea pedrosoi*](https://blast.ncbi.nlm.nih.gov/Blast.cgi#alnHdr_915073251) | 1e-177 | 96 | 52 | XP_013281868 |
| [Mannosyltransferase](https://blast.ncbi.nlm.nih.gov/Blast.cgi#alnHdr_1089737296) | 35748-37074 (-) | 242 | [*Cordyceps fumosorosea*](https://blast.ncbi.nlm.nih.gov/Blast.cgi#alnHdr_1089737296) | 3e-80 | 95 | 66 | XP_018703989 |
| [Translin-associated protein X](https://blast.ncbi.nlm.nih.gov/Blast.cgi#alnHdr_814598568) | 37608-38415 (+) | 236 | [*Trichoderma arundinaceum*](https://blast.ncbi.nlm.nih.gov/Blast.cgi#alnHdr_1464597932) | 2e-89 | 94 | 58 | RFU78724 |

| C. adiposa (B) - Contig 4 | | | | | | | |
| --- | --- | --- | --- | --- | --- | --- | --- |
| Predicted gene function | Position on contig | Size (aa) | Closest Species Ortholog | E-value | % Coverage | % Identity | NCBI Accession Number |
| Dual specificity protein kinase pom1 | 7847-12169 (-) | 1440 | [*Colletotrichum graminicola*](https://blast.ncbi.nlm.nih.gov/Blast.cgi#alnHdr_827061431) | 0.0 | 99 | 56 | XP_008092293 |
| Peroxisomal membrane protein | 18236-19990 (+) | 512 | [*Fusarium nygamai*](https://blast.ncbi.nlm.nih.gov/Blast.cgi#alnHdr_1333026751) | 0.0 | 85 | 61 | PNP77172 |
| [Geranylgeranyl pyrophosphate synthase](https://blast.ncbi.nlm.nih.gov/Blast.cgi#alnHdr_1267069085) | 20329-21963 (-) | 544 | [*Lomentospora prolificans*](https://blast.ncbi.nlm.nih.gov/Blast.cgi#alnHdr_1315198120) | 0.0 | 76 | 68 | PKS07637 |
| [Centromere binding protein B](https://blast.ncbi.nlm.nih.gov/Blast.cgi#alnHdr_1069511040) | 25090-26524 (-) | 461 | [*Lomentospora prolificans*](https://blast.ncbi.nlm.nih.gov/Blast.cgi#alnHdr_1315198120) | 1e-108 | 56 | 72 | PKS09270 |
| [Diphosphomevalonate decarboxylase](https://blast.ncbi.nlm.nih.gov/Blast.cgi#alnHdr_814601236) | 28648-29956 (-) | 382 | [*Lomentospora prolificans*](https://blast.ncbi.nlm.nih.gov/Blast.cgi#alnHdr_1315198120) | 0.0 | 99 | 77 | PKS12276 |

| C. fimbriata (A) - Contig 226 | | | | | | | |
| --- | --- | --- | --- | --- | --- | --- | --- |
| Predicted gene function | Position on contig | Size (aa) | Closest Species Ortholog | E-value | % Coverage | % Identity | NCBI Accession Number |
| [Glycylpeptide N-tetradecanoyltransferase](https://blast.ncbi.nlm.nih.gov/Blast.cgi#alnHdr_1267071815) | 42-1936 (+) | 567 | [*Ophiocordyceps australis*](https://blast.ncbi.nlm.nih.gov/Blast.cgi#alnHdr_1267094353) | 0.0 | 85 | 64 | PHH66240 |
| Hypothetical | 2493-5369 (+) | 958 | [*Hypoxylon sp. CO27-5*](https://blast.ncbi.nlm.nih.gov/Blast.cgi#alnHdr_1190898745) | 0.0 | 99 | 43 | OTA89062 |
| Hypothetical | 5784-8639 (+) | 842 | [*Colletotrichum higginsianum*](https://blast.ncbi.nlm.nih.gov/Blast.cgi#alnHdr_380492974) | 0.0 | 87 | 45 | CCF34210 |
| [Cytochrome b5 reductase](https://blast.ncbi.nlm.nih.gov/Blast.cgi#alnHdr_1267071814) | 8832-9824 (-) | 330 | [*Pochonia chlamydosporia*](https://blast.ncbi.nlm.nih.gov/Blast.cgi#alnHdr_1069535767) | 3e-95 | 100 | 52 | XP_018140598 |
| Hypothetical | 12054-15083 (+) | 1009 | [*Microdochium bolleyi*](https://blast.ncbi.nlm.nih.gov/Blast.cgi#alnHdr_1000222292) | 1e-76 | 63 | 38 | KXJ91297 |
| [putative J domain-containing protein](https://blast.ncbi.nlm.nih.gov/Blast.cgi#alnHdr_1267071827) | 16716-18481 (+) | 518 | [*Torrubiella hemipterigena*](https://blast.ncbi.nlm.nih.gov/Blast.cgi#alnHdr_729186369) | 0.0 | 99 | 68 | CEJ85347 |
| [squalene synthase](https://blast.ncbi.nlm.nih.gov/Blast.cgi#alnHdr_814598564) | 19683-21383 (+) | 470 | [*Scytalidium lignicola*](https://blast.ncbi.nlm.nih.gov/Blast.cgi#alnHdr_1464404747) | 0.0 | 93 | 63 | RFU24322 |
| [Alkaline protease 1](https://blast.ncbi.nlm.nih.gov/Blast.cgi#alnHdr_814598565) | 22483-23946 (-) | 487 | [*Scedosporium apiospermum*](https://blast.ncbi.nlm.nih.gov/Blast.cgi#alnHdr_1027069126) | 3e-173 | 79 | 66 | XP_016641262 |
| [Delta (24)-sterol reductase](https://blast.ncbi.nlm.nih.gov/Blast.cgi#alnHdr_1267071833) | 28749-30341 (-) | 501 | [*Phialocephala scopiformis*](https://blast.ncbi.nlm.nih.gov/Blast.cgi#alnHdr_1069196090) | 0.0 | 97 | 52 | XP_018062073 |
| Hypothetical | 32793-34731 (+) | 542 | [*Colletotrichum higginsianum*](https://blast.ncbi.nlm.nih.gov/Blast.cgi#alnHdr_1069498898) | 2e-105 | 96 | 35 | XP_018159040 |
| [GPI mannosyltransferase 1](https://blast.ncbi.nlm.nih.gov/Blast.cgi#alnHdr_1267071819) | 35720-36841 (-) | 347 | [*Colletotrichum higginsianum*](https://blast.ncbi.nlm.nih.gov/Blast.cgi#alnHdr_380492975) | 2e-155 | 98 | 67 | CCF34211 |
| [Translin-associated protein X](https://blast.ncbi.nlm.nih.gov/Blast.cgi#alnHdr_814598568) | 37365-38302 (+) | 267 | [*Fusarium pseudograminearum*](https://blast.ncbi.nlm.nih.gov/Blast.cgi#alnHdr_685858915) | 5e-97 | 97 | 57 | XP_009256490 |

| C. fimbriata (B) - Contig 106 | | | | | | | |
| --- | --- | --- | --- | --- | --- | --- | --- |
| Predicted gene function | Position on contig | Size (aa) | Closest Species Ortholog | E-value | % Coverage | % Identity | NCBI Accession Number |
| [Dual specificity protein kinase pom1](https://blast.ncbi.nlm.nih.gov/Blast.cgi#alnHdr_1267069088) | 7784-12406 (-) | 1540 | [*Colletotrichum higginsianum*](https://blast.ncbi.nlm.nih.gov/Blast.cgi#alnHdr_1069505056) | 0.0 | 99 | 51 | XP_018156292 |
| [Peroxisomal membrane protein](https://blast.ncbi.nlm.nih.gov/Blast.cgi#alnHdr_814601699) | 15787-17686 (+) | 552 | [*Fusarium verticillioides*](https://blast.ncbi.nlm.nih.gov/Blast.cgi#alnHdr_1092971283) | 0.0 | 84 | 58 | XP_018746623 |
| [Geranylgeranyl pyrophosphate synthase](https://blast.ncbi.nlm.nih.gov/Blast.cgi#alnHdr_1267069085) | 18268-20088 (-) | 606 | [*Lomentospora prolificans*](https://blast.ncbi.nlm.nih.gov/Blast.cgi#alnHdr_1315198120) | 0.0 | 61 | 72 | PKS07637 |
| [Cell division control protein 14](https://blast.ncbi.nlm.nih.gov/Blast.cgi#alnHdr_1267069082) | 23155-24170 (+) | 294 | [*Trichoderma asperellum*](https://blast.ncbi.nlm.nih.gov/Blast.cgi#alnHdr_1383953305) | 5e-138 | 99 | 71 | XP_024760524 |

| D. virescens (A) - Contig 263 | | | | | | | |
| --- | --- | --- | --- | --- | --- | --- | --- |
| Predicted gene function | Position on contig | Size (aa) | Closest Species Ortholog | E-value | % Coverage | % Identity | NCBI Accession Number |
| [Cytochrome b5 reductase 4](https://blast.ncbi.nlm.nih.gov/Blast.cgi#alnHdr_1373769672) | 2647-3720 (-) | 357 | [*Fusarium graminearum*](https://blast.ncbi.nlm.nih.gov/Blast.cgi#alnHdr_1246318085) | 3e-98 | 100 | 49 | XP_011328367 |
| Hypothetical | 8827-11622 (+) | 931 | [*Colletotrichum orbiculare*](https://blast.ncbi.nlm.nih.gov/Blast.cgi#alnHdr_477536195) | 8e-90 | 70 | 38 | ENH87669 |
| [putative J domain-containing protein](https://blast.ncbi.nlm.nih.gov/Blast.cgi#alnHdr_1267071827) | 14435-16312 (+) | 509 | [*Ophiocordyceps australis*](https://blast.ncbi.nlm.nih.gov/Blast.cgi#alnHdr_1267094372) | 0.0 | 100 | 64 | PHH66259 |
| [squalene synthase](https://blast.ncbi.nlm.nih.gov/Blast.cgi#alnHdr_1267071811) | 19502-21287 (+) | 474 | [*Neurospora crassa*](https://blast.ncbi.nlm.nih.gov/Blast.cgi#alnHdr_759000361) | 0.0 | 93 | 67 | XP_011395225 |
| [Alkaline protease 1](https://blast.ncbi.nlm.nih.gov/Blast.cgi#alnHdr_814598565) | 22634-23985 (-) | 430 | [*Scedosporium apiospermum*](https://blast.ncbi.nlm.nih.gov/Blast.cgi#alnHdr_1027069126) | 0.0 | 92 | 69 | XP_016641262 |

| D. virescens (B) - Contig 152 | | | | | | | |
| --- | --- | --- | --- | --- | --- | --- | --- |
| Predicted gene function | Position on contig | Size (aa) | Closest Species Ortholog | E-value | % Coverage | % Identity | NCBI Accession Number |
| [Dual specificity protein kinase pom1](https://blast.ncbi.nlm.nih.gov/Blast.cgi#alnHdr_1267069088) | 5812-10080 (-) | 1422 | [*Scedosporium apiospermum*](https://blast.ncbi.nlm.nih.gov/Blast.cgi#alnHdr_1027073664) | 0.0 | 99 | 55 | XP_016638384 |
| [Peroxisomal membrane protein PEX29](https://blast.ncbi.nlm.nih.gov/Blast.cgi#alnHdr_814601699) | 17665-19032 (+) | 455 | [*Trichoderma longibrachiatum*](https://blast.ncbi.nlm.nih.gov/Blast.cgi#alnHdr_1373372729) | 0.0 | 92 | 61 | PTB74644 |
| [Geranylgeranyl pyrophosphate synthase](https://blast.ncbi.nlm.nih.gov/Blast.cgi#alnHdr_1267069085) | 19676-21235 (-) | 519 | [*Lomentospora prolificans*](https://blast.ncbi.nlm.nih.gov/Blast.cgi#alnHdr_1315198120) | 0.0 | 64 | 73 | PKS07637 |
| [Cell division control protein 14](https://blast.ncbi.nlm.nih.gov/Blast.cgi#alnHdr_814601697) | 24930-25982 (+) | 321 | [*Colletotrichum chlorophyti*](https://blast.ncbi.nlm.nih.gov/Blast.cgi#alnHdr_1128257939) | 7e-134 | 99 | 66 | OLN86487 |
| Hypothetical | 26244-28001 (-) | 554 | [*Aspergillus sydowii*](https://blast.ncbi.nlm.nih.gov/Blast.cgi#alnHdr_1111885829) | 0.0 | 99 | 54 | OJJ54318 |

| E. polonica (A) - Contig 419 | | | | | | | |
| --- | --- | --- | --- | --- | --- | --- | --- |
| Predicted gene function | Position on contig | Size (aa) | Closest Species Ortholog | E-value | % Coverage | % Identity | NCBI Accession Number |
| Hypothetical | 3799-4923 (-) | 374 | [*Fusarium graminearum*](https://blast.ncbi.nlm.nih.gov/Blast.cgi#alnHdr_1246318085) | 7e-91 | 100 | 47 | PCD25687 |
| Hypothetical | 10059-12800 (+) | 913 | [*Colletotrichum orbiculare*](https://blast.ncbi.nlm.nih.gov/Blast.cgi#alnHdr_477536195) | 2e-84 | 72 | 37 | ENH87669 |
| [putative J domain-containing protein](https://blast.ncbi.nlm.nih.gov/Blast.cgi#alnHdr_1267071827) | 15364-17264 (+) | 509 | [*Drechmeria coniospora*](https://blast.ncbi.nlm.nih.gov/Blast.cgi#alnHdr_1057461794) | 0.0 | 99 | 65 | ODA79461 |
| [putative squalene synthase](https://blast.ncbi.nlm.nih.gov/Blast.cgi#alnHdr_1267071811) | 19661-21387 (+) | 466 | [*Neurospora tetrasperma*](https://blast.ncbi.nlm.nih.gov/Blast.cgi#alnHdr_698992596) | 0.0 | 99 | 64 | XP_009843279 |
| [Alkaline protease 1](https://blast.ncbi.nlm.nih.gov/Blast.cgi#alnHdr_814598565) | 22704-24083 (-) | 433 | [*Scedosporium apiospermum*](https://blast.ncbi.nlm.nih.gov/Blast.cgi#alnHdr_1027069126) | 0.0 | 91 | 68 | XP_016641262 |

| E. polonica (B) - Contig 150 | | | | | | | |
| --- | --- | --- | --- | --- | --- | --- | --- |
| Predicted gene function | Position on contig | Size (aa) | Closest Species Ortholog | E-value | % Coverage | % Identity | NCBI Accession Number |
| [Dual specificity protein kinase pom1](https://blast.ncbi.nlm.nih.gov/Blast.cgi#alnHdr_1267069088) | 6810-11075 (-) | 1421 | [*Lomentospora prolificans*](https://blast.ncbi.nlm.nih.gov/Blast.cgi#alnHdr_1315198122) | 0.0 | 99 | 54 | PKS07639 |
| [Peroxisomal membrane protein PEX29](https://blast.ncbi.nlm.nih.gov/Blast.cgi#alnHdr_814601699) | 17891-19255 (+) | 454 | [*Trichoderma longibrachiatum*](https://blast.ncbi.nlm.nih.gov/Blast.cgi#alnHdr_1373372729) | 0.0 | 92 | 61 | PTB74644 |
| [Geranylgeranyl pyrophosphate synthase](https://blast.ncbi.nlm.nih.gov/Blast.cgi#alnHdr_1267069085) | 19817-21352 (-) | 511 | [*Lomentospora prolificans*](https://blast.ncbi.nlm.nih.gov/Blast.cgi#alnHdr_1315198122) | 0.0 | 80 | 65 | PKS07637 |
| [Cell division control protein 14](https://blast.ncbi.nlm.nih.gov/Blast.cgi#alnHdr_814601697) | 24938-25963 (+) | 315 | [*Colletotrichum higginsianum*](https://blast.ncbi.nlm.nih.gov/Blast.cgi#alnHdr_1069505064) | 6e-135 | 99 | 68 | XP_018156296 |
| [Indoleamine 2,3-dioxygenase 1](https://blast.ncbi.nlm.nih.gov/Blast.cgi#alnHdr_1267067954) | 37987-39549 (-) | 500 | [*Colletotrichum simmondsii*](https://blast.ncbi.nlm.nih.gov/Blast.cgi#alnHdr_996611799) | 0.0 | 99 | 70 | KXH46294 |

| T. musarum (A) - Contig 23 | | | | | | | |
| --- | --- | --- | --- | --- | --- | --- | --- |
| Predicted gene function | Position on contig | Size (aa) | Closest Species Ortholog | E-value | % Coverage | % Identity | NCBI Accession Number |
| Hypothetical | 81-2024 (+) | 647 | [*Ophiocordyceps sp. 'camponoti-leonardi'*](https://blast.ncbi.nlm.nih.gov/Blast.cgi#alnHdr_1434078165) | 9e-123 | 100 | 38 | RDA86204 |
| [Cytochrome b5 reductase 4](https://blast.ncbi.nlm.nih.gov/Blast.cgi#alnHdr_1267071814) | 5283-6248 (-) | 321 | [*Verticillium longisporum*](https://blast.ncbi.nlm.nih.gov/Blast.cgi#alnHdr_913803165) | 5e-92 | 100 | 52 | CRK09967 |
| Hypothetical | 10053-12764 (+) | 903 | [*Pseudomassariella vexata*](https://blast.ncbi.nlm.nih.gov/Blast.cgi#alnHdr_1183433697) | 4e-81 | 70 | 37 | ORY57916 |
| [putative J domain-containing protein](https://blast.ncbi.nlm.nih.gov/Blast.cgi#alnHdr_814598563) | 14912-16837 (+) | 522 | [*Trichoderma arundinaceum*](https://blast.ncbi.nlm.nih.gov/Blast.cgi#alnHdr_1464597926) | 0.0 | 99 | 63 | RFU78718 |
| [putative squalene synthase](https://blast.ncbi.nlm.nih.gov/Blast.cgi#alnHdr_1267071811) | 19533-20922 (+) | 446 | [*Neurospora tetrasperma*](https://blast.ncbi.nlm.nih.gov/Blast.cgi#alnHdr_698992596) | 0.0 | 100 | 63 | XP_009853279 |
| [Alkaline protease 1](https://blast.ncbi.nlm.nih.gov/Blast.cgi#alnHdr_814598565) | 22254-23570 (-) | 438 | [*Scedosporium apiospermum*](https://blast.ncbi.nlm.nih.gov/Blast.cgi#alnHdr_1027069126) | 0.0 | 90 | 67 | XP_016641262 |

| T. musarum (B) - Contig 82 | | | | | | | |
| --- | --- | --- | --- | --- | --- | --- | --- |
| Predicted gene function | Position on contig | Size (aa) | Closest Species Ortholog | E-value | % Coverage | % Identity | NCBI Accession Number |
| [Dual specificity protein kinase pom1](https://blast.ncbi.nlm.nih.gov/Blast.cgi#alnHdr_1267069088) | 6589-11136 (-) | 1515 | [*Colletotrichum graminicola*](https://blast.ncbi.nlm.nih.gov/Blast.cgi#alnHdr_827061431) | 0.0 | 99 | 53 | XP_008092293 |
| Peroxisomal membrane protein | 17392-19239 (+) | 557 | *[Trichoderma harzianum](https://blast.ncbi.nlm.nih.gov/Blast.cgi#alnHdr_1308075954)* | 0.0 | 83 | 59 | PKK53072 |
| [Geranylgeranyl pyrophosphate synthase](https://blast.ncbi.nlm.nih.gov/Blast.cgi#alnHdr_1267069085) | 19745-21610 (-) | 621 | [*Lomentospora prolificans*](https://blast.ncbi.nlm.nih.gov/Blast.cgi#alnHdr_1315198120) | 0.0 | 55 | 76 | PKS07637 |
| [Cell division control protein 14](https://blast.ncbi.nlm.nih.gov/Blast.cgi#alnHdr_814601697) | 25029-26229 (+) | 318 | [*Colletotrichum chlorophyti*](https://blast.ncbi.nlm.nih.gov/Blast.cgi#alnHdr_1128257939) | 4e-138 | 99 | 67 | OLN86487 |
| Arsenite transmembrane transporter | 26357-28448 (-) | 642 | [*Stagonospora sp. SRC1lsM3a*](https://blast.ncbi.nlm.nih.gov/Blast.cgi#alnHdr_1028895387) | 0.0 | 98 | 57 | OAK97649 |
| [Indoleamine 2,3-dioxygenase 1](https://blast.ncbi.nlm.nih.gov/Blast.cgi#alnHdr_1267067954) | 31561-33115 (-) | 495 | *Colletotrichum simmondsii* | 0.0 | 99 | 69 | KXH46294 |
| Hypothetical | 34566-37147 (-) | 658 | *Fusarium avenaceum* | 3e-178 | 68 | 55 | KIL93736 |
