# Supplementary material for: Characterization of the Ergosterol Biosynthesis Pathway in Ceratocystidaceae
Source: J Fungi (Basel). 2021 Mar 22;7(3):237. doi: 10.3390/jof7030237 (PMC8004197; doi:10.3390/jof7030237)
Supplement: Supplementary file 1 [file jof-07-00237-s001.zip › Supplementary file S-2.docx]

| 3-h-3mg |  |  |  |  |
| --- | --- | --- | --- | --- |
| Strain | Number of Intron(s) | Position of intron(s) |  | Size |
|  |  | Start | Stop |  |
| *A. xylerbori* | 0 | None | None | 3468 |
| *C. adiposa* | 0 | None | None | 3639 |
| *C. fagacearum* | 0 | None | None | 3603 |
| *D. virescens* | 0 | None | None | 3645 |
| *E. polonica* | 0 | None | None | 3645 |
| *H. moniliformis* | 0 | None | None | 3468 |
| *B. basicola* | 0 | None | None | 3483 |
| *T. punctulata* | 0 | None | None | 3486 |

Supplementary file S-2. Tables represent number, position and size of introns of the ERG genes in different *Ceratocystidaceae*.

| aa-CoA thiolase |  |  |  |  |
| --- | --- | --- | --- | --- |
| Strain | Number of Intron(s) | Position of intron(s) |  | Size |
|  |  | Start | Stop |  |
| *A. xylerbori* | 3 | 66 | 148 | 1459 |
|  | 3 | 199 | 304 |  |
|  | 3 | 378 | 453 |  |
| *C. adiposa* | 2 | 199 | 337 | 1459 |
|  | 2 | 1286 | 1459 |  |
| *C. fagacearum* | 2 | 1 | 40 | 1361 |
|  | 2 | 967 | 1163 |  |
| *D. virescens* | 2 | 1 | 381 | 1548 |
|  | 2 | 1327 | 1419 |  |
| *E. polonica* | 1 | 1 | 103 | 1455 |
|  | 1 | 1258 | 1452 |  |
| *H. moniliformis* | 3 | 1004 | 1067 | 1386 |
|  | 3 | 1141 | 1198 |  |
|  | 3 | 1249 | 1318 |  |
| *B. basicola* | 2 | 199 | 272 | 1434 |
|  | 2 | 1226 | 1322 | 1434 |
| *T. punctulata* | 2 | 199 | 499 | 1468 |
|  | 2 | 1427 | 1468 | 1468 |

| FFP |  |  |  |  |
| --- | --- | --- | --- | --- |
| Strain | Number of Intron(s) | Position of intron(s) |  | Size |
|  |  | Start | Stop |  |
| *A. xylerbori* | 1 | 117 | 236 | 1160 |
| *C. adiposa* | 1 | 925 | 1059 | 1185 |
| *C. fagacearum* | 1 | 127 | 254 | 1178 |
| *D. virescens* | 1 | 130 | 286 | 1210 |
| *E. polonica* | 1 | 925 | 1072 | 1198 |
| *H. moniliformis* | 1 | 125 | 210 | 1163 |
| *B. basicola* | 1 | 925 | 1042 | 1162 |
| *T. punctulata* | 1 | 925 | 1078 | 1204 |

| IPP |  |  |  |  |
| --- | --- | --- | --- | --- |
| Strain | Number of Intron(s) | Position of intron(s) |  | Size |
|  |  | Start | Stop |  |
| *A. xylerbori* | 2 | 206 | 323 | 980 |
|  | 2 | 663 | 738 |  |
| *C. adiposa* | 2 | 1 | 205 | 971 |
|  | 2 | 324 | 662 |  |
| *C. fagacearum* | 0 | None | None | 1 |
| *D. virescens* | 1 | 240 | 326 | 657 |
| *E. polonica* | 0 | None | None | 1 |
| *H. moniliformis* | 2 | 194 | 265 | 978 |
|  | 2 | 605 | 736 |  |
| *B. basicola* | 2 | 224 | 321 | 991 |
|  | 2 | 661 | 773 |  |
| *T. punctulata* | 2 | 240 | 304 | 1042 |
|  | 2 | 644 | 840 |  |

| MVA diphosphate decarboxylase |  |  |  |  |
| --- | --- | --- | --- | --- |
| Strain | Number of Intron(s) | Position of intron(s) |  | Size |
|  |  | Start | Stop |  |
| *A. xylerbori* | 1 | 63 | 284 | 1401 |
| *C. adiposa* | 1 | 1118 | 1319 | 1381 |
| *C. fagacearum* | 1 | 63 | 177 | 1294 |
| *D. virescens* | 1 | 66 | 150 | 1267 |
| *E. polonica* | 1 | 1118 | 1185 | 1250 |
| *H. moniliformis* | 0 | None | None | 1203 |
| *B. basicola* | 1 | 1118 | 1218 | 1280 |
| *T. punctulata* | 1 | 1118 | 1184 | 1246 |

| MVA Kinase |  |  |  |  |
| --- | --- | --- | --- | --- |
| Strain | Number of Intron(s) | Position of intron(s) |  | Size |
|  |  | Start | Stop |  |
| *A. xylerbori* | 1 | 217 | 281 | 1577 |
| *C. adiposa* | 1 | 1297 | 1388 | 1597 |
| *C. fagacearum* | 1 | 1294 | 1375 | 1624 |
| *D. virescens* | 1 | 220 | 416 | 1603 |
| *E. polonica* | 1 | 193 | 299 | 1586 |
| *H. moniliformis* | 1 | 1297 | 1414 | 1657 |
| *B. basicola* | 2 | 1 | 268 | 1846 |
|  | 2 | 1558 | 1621 |  |
| *T. punctulata* | 0 | None | None | 1582 |

| phosphoMVA Kinase |  |  |  |  |
| --- | --- | --- | --- | --- |
| Strain | Number of Intron(s) | Position of intron(s) |  | Size |
|  |  | Start | Stop |  |
| *A. xylerbori* | 1 | 261 | 326 | 1362 |
| *C. adiposa* | 1 | 270 | 331 | 1373 |
| *C. fagacearum* | 1 | 1055 | 1217 | 1477 |
| *D. virescens* | 1 | 261 | 327 | 1372 |
| *E. polonica* | 1 | 261 | 340 | 1385 |
| *H. moniliformis* | 1 | 1040 | 1091 | 1333 |
| *B. basicola* | 1 | 261 | 384 | 1430 |
| *T. punctulata* | 1 | 261 | 411 | 1459 |

| C24 Sterol Desaturase | |  |  |  |
| --- | --- | --- | --- | --- |
| Strain | Number of Intron(s) | Position of intron(s) |  | Size |
|  |  | Start | Stop |  |
| *A. xylerbori* | 4 | 158 | 222 | 1839 |
|  | 4 | 374 | 441 |  |
|  | 4 | 1407 | 1467 |  |
|  | 4 | 1753 | 1803 |  |
| *C. adiposa* | 4 | 37 | 96 | 1752 |
|  | 4 | 381 | 433 |  |
|  | 4 | 1399 | 1466 |  |
|  | 4 | 1618 | 1694 |  |
| *C. fagacearum* | 4 | 1 | 41 | 1795 |
|  | 4 | 240 | 345 |  |
|  | 4 | 1328 | 1423 |  |
|  | 4 | 1564 | 1563 |  |
| *D. virescens* | 4 | 173 | 243 | 1888 |
|  | 4 | 395 | 472 |  |
|  | 4 | 1438 | 1506 |  |
|  | 4 | 1791 | 1852 |  |
| *E. polonica* | 4 | 37 | 103 | 1897 |
|  | 4 | 388 | 471 |  |
|  | 4 | 1437 | 1506 |  |
|  | 4 | 1658 | 1725 |  |
| *H. moniliformis* | 3 | 321 | 390 | 1811 |
|  | 3 | 1356 | 1428 |  |
|  | 3 | 1580 | 1648 |  |
| *B. basicola* | 4 | 1 | 21 | 1818 |
|  | 4 | 306 | 380 |  |
|  | 4 | 1349 | 1407 |  |
|  | 4 | 1559 | 1655 |  |
| *T. punctulata* | 4 | 164 | 238 | 1878 |
|  | 4 | 390 | 468 |  |
|  | 4 | 1434 | 1502 |  |
|  | 4 | 1787 | 1842 |  |

| ERG1 |  |  |  |  |
| --- | --- | --- | --- | --- |
| Strain | Number of Intron(s) | Position of intron(s) |  | Size |
|  |  | Start | Stop |  |
| *A. xylerbori* | 0 | None | None | 1497 |
| *C. adiposa* | 0 | None | None | 1503 |
| *C. fagacearum* | 0 | None | None | 1521 |
| *D. virescens* | 0 | None | None | 1509 |
| *E. polonica* | 0 | None | None | 1493 |
| *H. moniliformis* | 0 | None | None | 1500 |
| *B. basicola* | 0 | None | None | 1089 |
| *T. punctulata* | 0 | None | None | 1497 |

| ERG2 |  |  |  |  |
| --- | --- | --- | --- | --- |
| Strain | Number of Intron(s) | Position of intron(s) |  | Size |
|  |  | Start | Stop |  |
| *A. xylerbori* | 1 | 365 | 515 | 880 |
| *C. adiposa* | 1 | 356 | 502 | 867 |
| *C. fagacearum* | 1 | 366 | 446 | 789 |
| *D. virescens* | 1 | 347 | 704 | 1069 |
| *E. polonica* | 1 | 366 | 710 | 1056 |
| *H. moniliformis* | 1 | 366 | 510 | 850 |
| *B. basicola* | 1 | 366 | 436 | 770 |
| *T. punctulata* | 1 | 344 | 490 | 855 |

| ERG3 |  |  |  |  |
| --- | --- | --- | --- | --- |
| Strain | Number of Intron(s) | Position of intron(s) |  | Size |
|  |  | Start | Stop |  |
| *A. xylerbori* | 1 | 788 | 839 | 1084 |
| *C. adiposa* | 1 | 788 | 849 | 1094 |
| *C. fagacearum* | 3 | 52 | 332 | 1112 |
|  | 3 | 462 | 542 |  |
|  | 3 | 872 | 973 |  |
| *D. virescens* | 1 | 785 | 900 | 1145 |
| *E. polonica* | 1 | 246 | 353 | 1137 |
| *H. moniliformis* | 1 | 246 | 314 | 1101 |
| *B. basicola* | 1 | 785 | 933 | 1181 |
| *T. punctulata* | 1 | 785 | 858 | 1103 |

| ERG4 |  |  |  |  |
| --- | --- | --- | --- | --- |
| Strain | Number of Intron(s) | Position of intron(s) |  | Size |
|  |  | Start | Stop |  |
| *A. xylerbori* | 1 | 563 | 622 | 1596 |
| *C. adiposa* | 0 | None | None | 854 |
| *C. fagacearum* | 1 | 1032 | 1094 | 1656 |
| *D. virescens* | 1 | 1005 | 1064 | 1796 |
| *E. polonica* | 1 | 563 | 335 | 1624 |
| *H. moniliformis* | 1 | 951 | 1014 | 1576 |
| *B. basicola* | 1 | 563 | 796 | 1821 |
| *T. punctulata* | 2 | 1 | 303 | 2001 |
|  | 2 | 853 | 907 |  |

| ERG5 |  |  |  |  |
| --- | --- | --- | --- | --- |
| Strain | Number of Intron(s) | Position of intron(s) |  | Size |
|  |  | Start | Stop |  |
| *A. xylerbori* | 4 | 158 | 222 | 1839 |
|  | 4 | 374 | 441 |  |
|  | 4 | 1407 | 1468 |  |
|  | 4 | 1753 | 1803 |  |
| *C. adiposa* | 4 | 37 | 96 | 1857 |
|  | 4 | 381 | 433 |  |
|  | 4 | 1399 | 1466 |  |
|  | 4 | 1618 | 1694 |  |
| *C. fagacearum* | 3 | 330 | 435 | 1885 |
|  | 3 | 1418 | 1513 |  |
|  | 3 | 1654 | 1722 |  |
| *D. virescens* | 4 | 173 | 243 | 1888 |
|  | 4 | 395 | 472 |  |
|  | 4 | 1438 | 1506 |  |
|  | 4 | 1791 | 1852 |  |
| *E. polonica* | 4 | 37 | 103 | 1897 |
|  | 4 | 388 | 471 |  |
|  | 4 | 1437 | 1506 |  |
|  | 4 | 1658 | 1725 |  |
| *H. moniliformis* | 3 | 321 | 390 | 1811 |
|  | 3 | 1356 | 1428 |  |
|  | 3 | 1580 | 1648 |  |
| *B. basicola* | 4 | 212 | 1021 | 2818 |
|  | 4 | 1306 | 1380 |  |
|  | 4 | 2346 | 2407 |  |
|  | 4 | 2599 | 2655 |  |
| *T. punctulata* | 4 | 164 | 238 | 1878 |
|  | 4 | 390 | 468 |  |
|  | 4 | 1434 | 1502 |  |
|  | 4 | 1787 | 1842 |  |

| ERG6 |  |  |  |  |
| --- | --- | --- | --- | --- |
| Strain | Number of Intron(s) | Position of intron(s) |  | Size |
|  |  | Start | Stop |  |
| *A. xylerbori* | 2 | 574 | 625 | 1250 |
|  | 2 | 983 | 1046 |  |
| *C. adiposa* | 2 | 205 | 283 | 1279 |
|  | 2 | 641 | 706 |  |
| *C. fagacearum* | 2 | 574 | 638 | 1313 |
|  | 2 | 996 | 1103 |  |
| *D. virescens* | 2 | 574 | 640 | 1303 |
|  | 2 | 998 | 1054 |  |
| *E. polonica* | 2 | 211 | 308 | 1304 |
|  | 2 | 666 | 731 |  |
| *H. moniliformis* | 2 | 574 | 626 | 1273 |
|  | 2 | 984 | 1069 |  |
| *B. basicola* | 2 | 211 | 293 | 1291 |
|  | 2 | 651 | 718 |  |
| *T. punctulata* | 2 | 211 | 283 | 1304 |
|  | 2 | 641 | 731 |  |

| ERG7 |  |  |  |  |
| --- | --- | --- | --- | --- |
| Strain | Number of Intron(s) | Position of intron(s) |  | Size |
|  |  | Start | Stop |  |
| *A. xylerbori* | 1 | 211 | 292 | 2293 |
| *C. adiposa* | 2 | 211 | 307 | 2368 |
|  | 2 | 2021 | 2080 |  |
| *C. fagacearum* | 3 | 211 | 278 | 2377 |
|  | 3 | 1458 | 1514 |  |
|  | 3 | 1992 | 2089 |  |
| *D. virescens* | 3 | 214 | -802 | 2079 |
|  | 3 | 1464 | 1511 |  |
|  | 3 | 1998 | 2079 |  |
| *E. polonica* | 3 | 301 | 368 | 2196 |
|  | 3 | 855 | 902 |  |
|  | 3 | 2082 | 2196 |  |
| *H. moniliformis* | 2 | 211 | 265 | 2322 |
|  | 2 | 1979 | 2034 |  |
| *B. basicola* | 2 | 289 | 368 | 2351 |
|  | 2 | 2082 | 2141 |  |
| *T. punctulata* | 2 | 298 | 350 | 2359 |
|  | 2 | 2064 | 2149 |  |

| ERG9 |  |  |  |  |
| --- | --- | --- | --- | --- |
| Strain | Number of Intron(s) | Position of intron(s) |  | Size |
|  |  | Start | Stop |  |
| *A. xylerbori* | 1 | 57 | 556 | 1889 |
| *C. adiposa* | 1 | 57 | 612 | 1951 |
| *C. fagacearum* | 3 | 57 | 114 | 1701 |
|  | 3 | 172 | 324 |  |
|  | 3 | 1490 | 1566 |  |
| *D. virescens* | 2 | 57 | 351 | 1786 |
|  | 2 | 1562 | 1627 |  |
| *E. polonica* | 2 | 57 | 316 | 1727 |
|  | 2 | 1524 | 1589 |  |
| *H. moniliformis* | 1 | 57 | 389 | 1743 |
| *B. basicola* | 2 | 57 | 216 | 1608 |
|  | 2 | 1409 | 1476 |  |
| *T. punctulata* | 2 | 57 | 299 | 1696 |
|  | 2 | 1507 | 1575 |  |

| ERG24 |  |  |  |  |
| --- | --- | --- | --- | --- |
| Strain | Number of Intron(s) | Position of intron(s) |  | Size |
|  |  | Start | Stop |  |
| *A. xylerbori* | 3 | 60 | 182 | 1681 |
|  | 3 | 474 | 532 |  |
|  | 3 | 547 | 590 |  |
| *C. adiposa* | 0 | None | None | 984 |
| *C. fagacearum* | 2 | 1092 | 1218 | 1667 |
|  | 2 | 1479 | 1581 |  |
| *D. virescens* | 0 | None | None | 984 |
| *E. polonica* | 2 | 1092 | 1228 | 1567 |
|  | 2 | 1426 | 1567 |  |
| *H. moniliformis* | 1 | 60 | 124 | 1514 |
| *B. basicola* | 1 | 301 | 369 | 1460 |
| *T. punctulata* | 2 | 60 | 165 | 1623 |
|  | 2 | 471 | 532 |  |

| ERG25 |  |  |  |  |
| --- | --- | --- | --- | --- |
| Strain | Number of Intron(s) | Position of intron(s) |  | Size |
|  |  | Start | Stop |  |
| *A. xylerbori* | 2 | 1 | 190 | 1164 |
|  | 2 | 441 | 516 |  |
| *C. adiposa* | 1 | 649 | 734 | 965 |
| *C. fagacearum* | 3 | 15 | 258 | 1381 |
|  | 3 | 365 | 443 |  |
|  | 3 | 588 | 733 |  |
| *D. virescens* | 3 | 15 | 113 | 1189 |
|  | 3 | 220 | 324 |  |
|  | 3 | 469 | 531 |  |
| *E. polonica* | 3 | 15 | 88 | 1164 |
|  | 3 | 195 | 295 |  |
|  | 3 | 440 | 516 |  |
| *H. moniliformis* | 2 | 15 | 77 | 1039 |
|  | 2 | 328 | 391 |  |
| *B. basicola* | 3 | 15 | 88 | 1138 |
|  | 3 | 195 | 261 |  |
|  | 3 | 406 | 490 |  |
| *T. punctulata* | 3 | 15 | 168 | 1238 |
|  | 3 | 275 | 367 |  |
|  | 3 | 512 | 590 |  |

| ERG27 |  |  |  |  |
| --- | --- | --- | --- | --- |
| Strain | Number of Intron(s) | Position of intron(s) |  | Size |
|  |  | Start | Stop |  |
| *A. xylerbori* | 1 | 1376 | 1444 | 1512 |
| *C. adiposa* | 1 | 69 | 122 | 1464 |
| *C. fagacearum* | 2 | 69 | 126 | 1628 |
|  | 2 | 707 | 818 |  |
| *D. virescens* | 2 | 790 | 858 | 1613 |
|  | 2 | 1400 | 1545 | 1613 |
| *E. polonica* | 2 | 69 | 238 | 1652 |
|  | 2 | 780 | 863 | 1652 |
| *H. moniliformis* | 2 | 817 | 870 | 1452 |
|  | 2 | 1319 | 1381 | 1452 |
| *B. basicola* | 2 | 69 | 152 | 1608 |
|  | 2 | 694 | 798 | 1608 |
| *T. punctulata* | 1 | 773 | 896 | 1433 |

| ERG11 | | | | | |
| --- | --- | --- | --- | --- | --- |
| Strain | Number of Intron(s) | Position of intron(s) | | Size of introns | Size of the gene |
|  |  | Start | Stop |  |  |
| *B. basicola* | 1 | 258 | 329 | 71 | 1664 |
| *Davisoniella* | 1 | 1344 | 1406 | 62 | 1664 |
| *C. manginecans* | 1 | 1335 | 1406 | 71 | 1664 |
| *A. xylerbori* | 1 | 258 | 333 | 75 | 1674 |
| *E. polonica* | 1 | 1341 | 1407 | 66 | 1665 |
| *C. adiposa* | 1 | 1348 | 1417 | 69 | 1675 |
| *T. punctulata* | 1 | 1344 | 1413 | 69 | 1671 |
| *H. moniliformis* | 1 | 1341 | 1413 | 72 | 1708 |
| *B. fagacearum* | 1 | 258 | 330 | 72 | 1674 |

| ERG13 | | | | | |
| --- | --- | --- | --- | --- | --- |
| Strain | Number of Intron(s) | Position of intron(s) | | Size of introns | Size of the gene |
|  |  | Start | Stop |  |  |
| *B. basicola* | 3 | 138 | 200 | 62 | 1655 |
|  |  | 1265 | 1425 | 160 | 1655 |
|  |  | 1529 | 1595 | 66 | 1655 |
| *Davisoniella* | 3 | 60 | 137 | 77 | 1925 |
|  |  | 241 | 611 | 370 | 1925 |
|  |  | 1634 | 1727 | 93 | 1925 |
| *C. manginecans* | 3 | 60 | 202 | 142 | 1926 |
|  |  | 306 | 657 | 351 | 1926 |
|  |  | 1704 | 1788 | 84 | 1926 |
| *A. xylerbori* | 2 | 63 | 198 | 135 | 1821 |
|  |  | 302 | 643 | 341 | 1821 |
| *E. polonica* | 3 | 60 | 133 | 73 | 1885 |
|  |  | 237 | 652 | 415 | 1885 |
|  |  | 1679 | 1747 | 68 | 1885 |
| *C. adiposa* | 2 | 63 | 215 | 152 | 1851 |
|  |  | 319 | 673 | 354 | 1851 |
| *T. punctulata* | 3 | 60 | 131 | 71 | 1797 |
|  |  | 235 | 560 | 325 | 1797 |
|  |  | 1601 | 1659 | 58 | 1797 |
| *H. moniliformis* | 2 | 63 | 182 | 119 | 1806 |
|  |  | 286 | 628 | 342 | 1806 |
| *B. fagacearum* | 2 | 63 | 224 | 161 | 1818 |
|  |  | 328 | 640 | 312 | 1818 |
