# Supplementary material for: Characterization of the Ergosterol Biosynthesis Pathway in Ceratocystidaceae
Source: J Fungi (Basel). 2021 Mar 22;7(3):237. doi: 10.3390/jof7030237 (PMC8004197; doi:10.3390/jof7030237)
Supplement: Supplementary file 1 [file jof-07-00237-s001.zip › Supplementary file S-3.docx]

**Supplementary Table S-3**. The tables (A-I) below show the top tblastn hits NCBI’s non-redundant nucleotide database for each of the Ceratocystidaceae genes involved in mevalonate (MVA) as well as ergosterol biosynthesis pathways. The blast scores (E-value, percent sequence identity and coverage) and the accession number of the top hits are indicated.

| ***A. xylebori*** | | | | | | | | | |
| --- | --- | --- | --- | --- | --- | --- | --- | --- | --- |
| Pathway | Contig Number | Location | E-value | % coverage | % identity | BLAST sequence description | NCBI Accession Number | Closest species ortholog | Predicted gene function |
| MVA | PCDO01000004 | 1382306-1385773 | 0.0 | 99 | 69 | hydroxy methyl glutaryl CoA reductase | OLN85448 | *Colletotrichum chlorophyti* | 3-hydroxy-3-methylglutaryl-CoA reductase |
|  | PCDO01000006 | 802137-803595 | 0.0 | 99 | 78 | acetyl-CoA C-acetyltransferas | KPA40141 | *Fusarium langsethiae* | Acetoacetyl-CoA thiolase (ERG 10) |
|  | PCDO01000012 | 663832-664991 | 4e-173 | 98 | 69 | Farnesyl pyrophosphate synthetase | XP_011326434 | *Fusarium graminearum* | Farnesyl pyrophosphate synthetase (ERG 20) |
|  | PCDO01000013 | 88959-90780 | 0.0 | 99 | 76 | hydroxymethylglutaryl CoA synthetase | PSR76629 | *Coniella lustricola* | hydroxymethylglutaryl CoA synthetase (ERG 13) |
|  | PCDO01000002 | 1668199-1669178 | 4e-143 | 93 | 79 | isopentenyl-diphosphate delta-isomerase | KXJ92177 | *Microdochium bolleyi* | isopentenyl-diphosphate delta-isomerase |
|  | PCDO01000008 | 1489174-1490574 | 0.0 | 80 | 76 | Diphosphomevalonate decarboxylase | PKS12276 | *Lomentospora prolificans* | Diphosphomevalonate decarboxylase |
|  | PCDO01000012 | 949339-950915 | 0.0 | 97 | 72 | Mevalonate kinase | XP_023428990 | [*Fusarium*](https://blast.ncbi.nlm.nih.gov/Blast.cgi#alnHdr_685854856) *fujikuroi* | Mevalonate kinase (ERG 12) |
|  | PCDO01000001 | 4120203-4121564 | 2e-8 | 87 | 65 | [phosphomevalonate kinase](https://blast.ncbi.nlm.nih.gov/Blast.cgi#alnHdr_808376609) | [XM_012337726.1](https://www.ncbi.nlm.nih.gov/nucleotide/XM_012337726.1?report=genbank&log$=nucltop&blast_rank=1&RID=GUFVCVUU015) | [*Pseudozyma hubeiensis*](https://blast.ncbi.nlm.nih.gov/Blast.cgi#alnHdr_808376609) | Phosphomevalonate kinase (ERG 8) |
| Sterol | PCDO01000005 | 534268-535764 | 0 | 99 | 64 | Squalene epoxidase | KFH43726 | *Acremonium chrysogenum* | Squalene epoxidase (ERG 1) |
|  | PCDO01000007 | 1446204-1447715 | 1e-151 | 99 | 47 | 3-keto steroid reductase | XP_018157938 | *Colletotrichum higginsianum* | 3-keto sterol reductase (ERG 27) |
|  | PCDO01000004 | 172832-173381 | 7e-73 | 97 | 65 | Ergosterol biosynthesis protein 28 | XP_022474034 | *Colletotrichum orchidophilum* | Ergosterol biosynthesis protein 28 (ERG 28) |
|  | PCDO01000001 | 2816317-2817998 | 0.0 | 99 | 65 | [Delta(14)-sterol reductase](https://blast.ncbi.nlm.nih.gov/Blast.cgi#alnHdr_315055184) | XP_016642425 | *Scedosporium apiospermum* | C-[(14)-sterol reductase](https://blast.ncbi.nlm.nih.gov/Blast.cgi#alnHdr_315055184) (ERG 24) |
|  | PCDO01000005 | 219025-220164 | 2e-175 | 100 | 76 | [C-4 methylsterol oxidase](https://blast.ncbi.nlm.nih.gov/Blast.cgi#alnHdr_1069534612) | XP_016640914 | *Scedosporium apiospermum* | C4 sterol methyl oxidase (ERG 25) |
|  | PCDO01000006 | 1683096-1684179 | 2e-91 | 49 | 79 | C-5 sterol desaturase | [XM_014096448.1](https://www.ncbi.nlm.nih.gov/nucleotide/XM_014096448.1?report=genbank&log$=nucltop&blast_rank=1&RID=GUBWM9F6014) | [*Trichoderma virens*](https://blast.ncbi.nlm.nih.gov/Blast.cgi#alnHdr_927412183) | C5 sterol desaturase (ERG 3) |
|  | PCDO01000001 | 3974436-3975315 | 3e-112 | 90 | 71 | [C-8 sterol isomerase](https://blast.ncbi.nlm.nih.gov/Blast.cgi#alnHdr_1087780056) | XP_003649780 | *Thielavia terrestris* | C8 sterol isomerase (ERG2) |
|  | PCDO01000001 |  | 0.0 | 73 | 78 | [Delta(24(24(1)))-sterol reductase](https://blast.ncbi.nlm.nih.gov/Blast.cgi#alnHdr_697069466) | [XM_009651622.1](https://www.ncbi.nlm.nih.gov/nucleotide/XM_009651622.1?report=genbank&log$=nucltop&blast_rank=1&RID=GUBWM9F6014) | [*Verticillium dahliae*](https://blast.ncbi.nlm.nih.gov/Blast.cgi#alnHdr_697069466) | C-24 [sterol reductase](https://blast.ncbi.nlm.nih.gov/Blast.cgi#alnHdr_697069466) (ERG4) |
|  | PCDO01000014 | 101851-103689 | 3e-80 | 41 | 75 | Cytochrome P-450 | [XM_007711043.1](https://www.ncbi.nlm.nih.gov/nucleotide/XM_007711043.1?report=genbank&log$=nucltop&blast_rank=1&RID=GUBWM9F6014) | [*Bipolaris zeicola*](https://blast.ncbi.nlm.nih.gov/Blast.cgi#alnHdr_628193518) | Cytochrome P-450 (ERG 5) |
|  | PCDO01000002 | 2000454-2002746 | 0.0 | 99 | 72 | Lanosterol synthase | PKS07236 | *Lomentospora prolificans* | Lanosterol synthase (ERG 7) |
|  | PCDO01000001 | 791258-793146 | 0.0 | 98 | 63 | Squalene synthase | XP_001905353 | *Podospora anserina* | Squalene synthase (ERG 9) |
|  | PCDO01000018 | 166730-167979 | 0.0 | 99 | 84 | [sterol 24-C-methyltransferase](https://blast.ncbi.nlm.nih.gov/Blast.cgi#alnHdr_758201340) | XP_018157428 | *Colletotrichym higginsianum* | C24 sterol methyltransferase (ERG 6) |
|  | PCDO01000001 | 1896760-1898434 | 0.0 | 99 | 72 | proliferating cell nuclear antigen | PKS10573 | *Lomentospora prolificans* | C14 demethylase (ERG 11) |

| ***B. fagacearum*** | | | | | | | | | |
| --- | --- | --- | --- | --- | --- | --- | --- | --- | --- |
| Pathway | Contig Number | Location | E-value | % Similarity | % Coverage | BLAST sequence description | NCBI Accession Number | Closest species ortholog | Predicted gene function |
| MVA | MKGJ01000135 | 34709-38311 | 0.0 | 96 | 69 | 3-hydroxy-3-methylglutaryl-CoA reductase | OLN85448 | *Colletotrichum chlorophyti* | 3-hydroxy-3-methylglutaryl-CoA reductase |
|  | MKGJ01000069 | 57657-59136 | 0.0 | 99 | 71 | acetyl-CoA C-acetyltransferase | [XM_003054575.1](https://www.ncbi.nlm.nih.gov/nucleotide/XM_003054575.1?report=genbank&log$=nucltop&blast_rank=1&RID=GUE4V1XG015) | *Fusarium langsethiae* | Acetoacetyl-CoA thiolase (ERG 10) |
|  | MKGJ01000599 | 11261-12438 | 1e-178 | 98 | 68 | [farnesyl pyrophosphate synthetase](https://blast.ncbi.nlm.nih.gov/Blast.cgi#alnHdr_1069609557) | PNY27092 | *Tolypocladium capitatum* | Farnesyl pyrophosphate synthetase (ERG 20) |
|  | MKGJ01000008 | 93102-94920 | 0.0 | 99 | 77 | [hydroxymethylglutaryl CoA synthase](https://blast.ncbi.nlm.nih.gov/Blast.cgi#alnHdr_589101550) | XP_957561 | *Neurospora crassa* | hydroxymethylglutaryl CoA synthetase (ERG 13) |
|  | MKGJ01000339 | 21334-22400 | 1e-143 | 93 | 78 | isopentenyl-diphosphate delta-isomerase | KXJ92177 | *Microdochium bolleyi* | isopentenyl-diphosphate delta-isomerase |
|  | MKGJ01000165 | 28237-29589 | 0.0 | 99 | 75 | mevalonate pyrophosphate decarboxylase | PNH45718 | *Verticillium dahliae* | Diphosphomevalonate decarboxylase |
|  | MKGJ01000152 | 42713-44352 | 0.0 | 96 | 72 | Mevalonate kinase | KLO96633 | [*Fusarium*](https://blast.ncbi.nlm.nih.gov/Blast.cgi#alnHdr_685854856) *fujikuroi* | Mevalonate kinase (ERG 12) |
|  | MKGJ01000004 | 161873-163231 | 1e-7 | 84 | 89 | [phosphomevalonate kinase](https://blast.ncbi.nlm.nih.gov/Blast.cgi#alnHdr_808376609) | [XM_012337726.1](https://www.ncbi.nlm.nih.gov/nucleotide/XM_012337726.1?report=genbank&log$=nucltop&blast_rank=1&RID=GUFVCVUU015) | [*Pseudozyma hubeiensis*](https://blast.ncbi.nlm.nih.gov/Blast.cgi#alnHdr_808376609) | phosphomevalonate kinase (ERG 8) |
| Sterol | MKGJ01000030 | 41832-43315 | 3e-152 | 100 | 50 | Hypothetical | XP_008099856 | *Colletotrichum graminicola* | 3-keto sterol reductase (ERG 27) |
|  | MKGJ01000006 | 19623-20246 | 6e-54 | 97 | 65 | Ergosterol biosynthesis protein 28 | OLN97423 | *Colletotrichum chlorophyti* | Ergosterol biosynthesis protein 28 (ERG 28) |
|  | MKGJ01000408 | 14390-15562 | 5e-176 | 100 | 76 | [C-4 methylsterol oxidase](https://blast.ncbi.nlm.nih.gov/Blast.cgi#alnHdr_697083940) | XP_016640914 | *Scedosporium apiospermum* | C4 sterol methyl oxidase (ERG 25) |
|  | MKGJ01000001 | 106606-107702 | 1e-175 | 99 | 68 | C-5 sterol desaturase | XP_006965542 | *Trichoderma reesei* | C5 sterol desaturase (ERG 3) |
|  | MKGJ01000034 | 88311-89184 | 1e-57 | 71 | 80 | [C-8 sterol isomerase](https://blast.ncbi.nlm.nih.gov/Blast.cgi#alnHdr_389626796) | [XM_003711004.1](https://www.ncbi.nlm.nih.gov/nucleotide/XM_003711004.1?report=genbank&log$=nucltop&blast_rank=1&RID=GUFVCVUU015) | [*Magnaporthe oryzae*](https://blast.ncbi.nlm.nih.gov/Blast.cgi#alnHdr_389626796) | C8 sterol isomerase (ERG 2) |
|  | MKGJ01000380 | 5644-7331 | 0.0 | 86 | 77 | [Delta(24(24(1)))-sterol reductase](https://blast.ncbi.nlm.nih.gov/Blast.cgi#alnHdr_697069466) | [XM_009651622.1](https://www.ncbi.nlm.nih.gov/nucleotide/XM_009651622.1?report=genbank&log$=nucltop&blast_rank=1&RID=GUFVCVUU015) | [*Verticillium dahliae*](https://blast.ncbi.nlm.nih.gov/Blast.cgi#alnHdr_697069466) | C24 sterol reductase (ERG 4) |
|  | MKGJ01000169 | 26503-28391 | 0.0 | 99 | 79 | Cytochrome P-450 | OLN95588 | *Colletotrichum chlorophyti* | Cytochrome P-450 (ERG 5) |
|  | MKGJ01000191 | 39862-41657 | 0.0 | 99 | 66 | C-14 sterol reductase | XP_016642425 | *Scedosporium apiospermum* | C-14 sterol reductase (ERG 24) |
|  | MKGJ01000093 | 54508-55776 | 0.0 | 97 | 78 | [delta(24)-sterol C-methyltransferase](https://blast.ncbi.nlm.nih.gov/Blast.cgi#alnHdr_1328539265) | [XM_023578823.1](https://www.ncbi.nlm.nih.gov/nucleotide/XM_023578823.1?report=genbank&log$=nucltop&blast_rank=1&RID=GUE4V1XG015) | [*Fusarium fujikuroi*](https://blast.ncbi.nlm.nih.gov/Blast.cgi#alnHdr_1328539265) | C24 sterol methyltransferase (ERG 6) |
|  | MKGJ01000162 | 14814-17206 | 0.0 | 99 | 72 | Lanosterol synthase | XP_016640229 | *Scedosporium apiospermum* | Lanosterol synthase (ERG 7) |
|  | MKGJ01000380 | 2142-3978 | 0.0 | 98 | 63 | Squalene synthase | XP_011395224 | *Neurospora crassa* | Squalene synthase (ERG 9) |
|  | MKGJ01000012 | 67949-69623 | 0.0 | 99 | 73 | [14-alpha-demethylase](https://blast.ncbi.nlm.nih.gov/Blast.cgi#alnHdr_1069483388) | PKS10573 | *Lomentospora prolificans* | C14 demethylase (ERG 11) |

| ***C. fimbriata*** | | | | | | | | | |
| --- | --- | --- | --- | --- | --- | --- | --- | --- | --- |
| Pathway | Contig Number | Location | E-value | % Similarity | % Coverage | BLAST sequence description | NCBI Accession Number | Closest species ortholog | Predicted gene function |
| MVA | APWK02000033 | 28377-31877 | 0.0 | 99 | 69 | [hydroxy methyl glutaryl CoA reductase](https://blast.ncbi.nlm.nih.gov/Blast.cgi#alnHdr_985708128) | OLN85448 | *Colletotrichum chlorophyti* | 3-hydroxy-3-methylglutaryl-CoA reductase |
|  | APWK02000502 | 8320-9803 | 0.0 | 99 | 80 | acetyl-CoA acetyltransferase | GAP88710 | *Rosellinia necatrix* | Acetoacetyl-CoA thiolase (ERG 10) |
|  | APWK02000393 | 23900-25031 | 0.0 | 100 | 70 | Farnesyl pyrophosphate synthetase | PMD37588 | *Meliniomyces variabilis* | Farnesyl pyrophosphate synthetase (ERG 20) |
|  | APWK02000786 | 12477-14403 | 0.0 | 100 | 81 | hydroxymethylglutaryl CoA synthetase | ELA34245 | *Colletotrichum gloeosporioides* | hydroxymethylglutaryl CoA synthetase (ERG 13) |
|  | APWK02001056 | 131-1190 | 5e-133 | 99 | 68 | isopentenyl-diphosphate delta-isomerase | OTA63645 | *Hypoxylon sp.* | isopentenyl-diphosphate delta-isomerase |
|  | APWK02000666 | 403-1696 | 0.0 | 98 | 79 | [mevalonate pyrophosphate decarboxylase](https://blast.ncbi.nlm.nih.gov/Blast.cgi#alnHdr_589109814) | XP_016644508 | *Scedosporium apiospermum* | Diphosphomevalonate decarboxylase |
|  | APWK02000150 | 19623-21246 | 0.0 | 97 | 73 | Mevalonate kinase | KPA45901 | [*Fusarium*](https://blast.ncbi.nlm.nih.gov/Blast.cgi#alnHdr_685854856) *langsethiae* | Mevalonate kinase (ERG 12) |
|  | APWK02000821 | 13105-14581 | 3e-151 | 99 | 71 | phosphomevalonate kinase | POR33036 | *Tolypocladium paradoxum* | phosphomevalonate kinase (ERG 8) |
| Sterol | APWK02000276 | 9219-10846 | 7e-148 | 100 | 66 | 3-keto steroid reductase | ENH81517 | *Colletotrichum orbiculare* | 3-keto sterol reductase (ERG 27) |
|  | APWK02000059 | 41832-43315 | 2e-154 | 97 | 65 | Ergosterol biosynthesis protein 28 | XP_022474034 | *Colletotrichum orchidophilum* | Ergosterol biosynthesis protein 28 (ERG 28) |
|  | APWK02000733 | 17018-18657 | 0.0 | 99 | 67 | Delta (14)-sterol reductase | XP_016642425 | *Scedosporium apiospermum* | C-14-sterol reductase (ERG 24) |
|  | APWK02000374 | 33414-34794 | 6e-172 | 100 | 75 | [fatty acid hydroxylase](https://blast.ncbi.nlm.nih.gov/Blast.cgi#alnHdr_827056543) | XP_016640914 | *Scedosporium apiospermum* | C4 sterol methyl oxidase (ERG 25) |
|  | APWK02000226 | 27153-28728 | 2e-171 | 77 | 71 | delta24(24(1))-sterol reductase | XM_003050685.1 | *Nectria haematococca* | C24 sterol reductase (ERG 4) |
|  | APWK02000652 | 21810-23011 | 3e-110 | 80 | 77 | C5 sterol desaturase | [XM_014096448.1](https://www.ncbi.nlm.nih.gov/nucleotide/XM_014096448.1?report=genbank&log$=nucltop&blast_rank=1&RID=GUF4EDXZ014) | [*Trichoderma virens*](https://blast.ncbi.nlm.nih.gov/Blast.cgi#alnHdr_927412183) | C5 sterol desaturase (ERG 3) |
|  | APWK02000733 | 4621-5409 | 3e-51 | 80 | 47 | C8 sterol isomerase | [XM_006961378.1](https://www.ncbi.nlm.nih.gov/nucleotide/XM_006961378.1?report=genbank&log$=nucltop&blast_rank=1&RID=GUF4EDXZ014) | [*Trichoderma reesei*](https://blast.ncbi.nlm.nih.gov/Blast.cgi#alnHdr_589098834) | C8 sterol isomerase (ERG 2) |
|  | APWK02001147 | 4322-7794 | 0 | 82 | 75 | Cytochrome P-450 | OLN95588 | *Colletotrichum chlorophyti* | Cytochrome P-450 (ERG 5) |
|  | APWK02000708 | 12060-13372 | 2e-72 | 74 | 68 | [Delta(24)-sterol C-methyltransferase](https://blast.ncbi.nlm.nih.gov/Blast.cgi#alnHdr_1370882702) | [XM_024468559.1](https://www.ncbi.nlm.nih.gov/nucleotide/XM_024468559.1?report=genbank&log$=nucltop&blast_rank=1&RID=GUF4EDXZ014) | [*Pseudogymnoascus destructans*](https://blast.ncbi.nlm.nih.gov/Blast.cgi#alnHdr_1370882702) | C24 sterol methyltransferase (ERG 6) |
|  | APWK02000561 | 10484-12860 | 0.0 | 99 | 69 | Lanosterol synthase | XP_008098265 | *Colletotrichum graminicola* | Lanosterol synthase (ERG 7) |
|  | APWK02000226 | 26282-27982 | 0.0 | 98 | 60 | Squalene synthase | XP_011395224 | *Neurospora crassa* | Squalene synthase (ERG 9) |
|  | APWK02000385 | 7177-10548 | 6e-142 | 88 | 74 | proliferating cell nuclear antigen | [XM_008092297.1](https://www.ncbi.nlm.nih.gov/nucleotide/XM_008092297.1?report=genbank&log$=nucltop&blast_rank=1&RID=GUF4EDXZ014) | [*Colletotrichum graminicola*](https://blast.ncbi.nlm.nih.gov/Blast.cgi#alnHdr_827057745) | C14 demethylase (ERG 11) |

| ***C. manginecans*** | | | | | | | | | |
| --- | --- | --- | --- | --- | --- | --- | --- | --- | --- |
| Pathway | Contig Number | Location | E-value | % Similarity | % Coverage | BLAST sequence description | NCBI Accession Number | Closest species ortholog | Predicted gene function |
| MVA | JJRZ01000113 | 28210-31869 | 0.0 | 98 | 69 | [hydroxy methyl glutaryl CoA reductase](https://blast.ncbi.nlm.nih.gov/Blast.cgi#alnHdr_985708128) | OLN85448 | *Colletotrichum chlorophyti* | 3-hydroxy-3-methylglutaryl-CoA reductase |
|  | JJRZ01000073 | 49026-50138 | 0.0 | 97 | 82 | acetyl-CoA acetyltransferase | GAP88710 | *Rosellinia necatrix* | Acetoacetyl-CoA thiolase (ERG 10) |
|  | JJRZ01000074 | 27888-28808 | 0.0 | 100 | 70 | Farnesyl pyrophosphate synthetase | PMD37588 | *Meliniomyces variabilis* | Farnesyl pyrophosphate synthetase (ERG 20) |
|  | JJRZ01000072 | 91063-92112 | 0.0 | 100 | 81 | hydroxymethylglutaryl CoA synthetase | ELA34245 | *Colletotrichum gloeosporioides* | hydroxymethylglutaryl CoA synthetase (ERG 13) |
|  | JJRZ01000315 | 25025-25723 | e-133 | 99 | 68 | isopentenyl-diphosphate delta-isomerase | OTA63645 | *Hypoxylon sp.* | isopentenyl-diphosphate delta-isomerase |
|  | JJRZ01000097 | 31880-32911 | 0.0 | 9 | 79 | [mevalonate pyrophosphate decarboxylase](https://blast.ncbi.nlm.nih.gov/Blast.cgi#alnHdr_589109814) | XP_016644508 | *Scedosporium apiospermum* | Diphosphomevalonate decarboxylase |
|  | JJRZ01000022 | 137708-139000 | 0.0 | 97 | 73 | Mevalonate kinase | KPA45901 | [*Fusarium*](https://blast.ncbi.nlm.nih.gov/Blast.cgi#alnHdr_685854856) *langsethiae* | Mevalonate kinase (ERG 12) |
|  | JJRZ01000123 | 52081-53130 | 3e-151 | 99 | 71 | phosphomevalonate kinase | POR33036 | *Tolypocladium paradoxum* | phosphomevalonate kinase (ERG 8) |
| Sterol | JJRZ01000162 | 14512-15309 | 6e-138 | 100 | 66 | 3-keto steroid reductase | ENH81517 | *Colletotrichum orbiculare* | 3-keto sterol reductase (ERG 27) |
|  | JJRZ01000067 | 41832-43315 | 3e-55 | 98 | 66 | Ergosterol biosynthesis protein 28 | XP_022474034 | *Colletotrichum orchidophilum* | Ergosterol biosynthesis protein 28 (ERG 28) |
|  | JJRZ01000070 | 11020-12659 | 0.0 | 99 | 67 | Delta (14)-sterol reductase | XP_016642425 | *Scedosporium apiospermum* | C-14-sterol reductase (ERG 24) |
|  | JJRZ01000037 | 50074-50733 | 5e-145 | 100 | 75 | [fatty acid hydroxylase](https://blast.ncbi.nlm.nih.gov/Blast.cgi#alnHdr_827056543) | XP_016640914 | *Scedosporium apiospermum* | C4 sterol methyl oxidase (ERG 25) |
|  | JJRZ01000111 | 69046-70244 | 3e-110 | 79 | 78 | C5 sterol desaturase | [XM_014096448.1](https://www.ncbi.nlm.nih.gov/nucleotide/XM_014096448.1?report=genbank&log$=nucltop&blast_rank=1&RID=GUF4EDXZ014) | [*Trichoderma virens*](https://blast.ncbi.nlm.nih.gov/Blast.cgi#alnHdr_927412183) | C5 sterol desaturase (ERG 3) |
|  | JJRZ01000076 | 2501-3256 | 3e-51 | 80 | 47 | C8 sterol isomerase | [XM_006961378.1](https://www.ncbi.nlm.nih.gov/nucleotide/XM_006961378.1?report=genbank&log$=nucltop&blast_rank=1&RID=GUF4EDXZ014) | [*Trichoderma reesei*](https://blast.ncbi.nlm.nih.gov/Blast.cgi#alnHdr_589098834) | C8 sterol isomerase (ERG 2) |
|  | JJRZ01000004 | 16554-18206 | 2e-171 | 75 | 71 | delta24(24(1))-sterol reductase | XM_003050685.1 | *Nectria haematococca* | C24 sterol reductase (ERG 4) |
|  | JJRZ01000061 | 16580-18070 | 0 | 81 | 74 | Cytochrome P-450 | OLN95588 | *Colletotrichum chlorophyti* | Cytochrome P-450 (ERG 5) |
|  | JJRZ01000112 | 65159-65734 | 2e-72 | 74 | 68 | [Delta(24)-sterol C-methyltransferase](https://blast.ncbi.nlm.nih.gov/Blast.cgi#alnHdr_1370882702) | [XM_024468559.1](https://www.ncbi.nlm.nih.gov/nucleotide/XM_024468559.1?report=genbank&log$=nucltop&blast_rank=1&RID=GUF4EDXZ014) | [*Pseudogymnoascus destructans*](https://blast.ncbi.nlm.nih.gov/Blast.cgi#alnHdr_1370882702) | C24 sterol methyltransferase (ERG 6) |
|  | JJRZ01000256 | 8596-10308 | 0.0 | 97 | 70 | Lanosterol synthase | XP_008098265 | *Colletotrichum graminicola* | Lanosterol synthase (ERG 7) |
|  | JJRZ01000004 | 131507-131638 | 0.0 | 99 | 61 | Squalene synthase | XP_011395224 | *Neurospora crassa* | Squalene synthase (ERG 9) |
|  | JJRZ01000152 | 45683-47011 | 4e-112 | 88 | 74 | proliferating cell nuclear antigen | [XM_008092297](https://www.ncbi.nlm.nih.gov/nucleotide/XM_008092297.1?report=genbank&log$=nucltop&blast_rank=1&RID=GUF4EDXZ014) | [*Colletotrichum graminicola*](https://blast.ncbi.nlm.nih.gov/Blast.cgi#alnHdr_827057745) | C14 demethylase (ERG 11) |

| ***C. eucalypticola*** | | | | | | | | | |
| --- | --- | --- | --- | --- | --- | --- | --- | --- | --- |
| Pathway | Contig Number | Location | E-value | % Similarity | % Coverage | BLAST sequence description | NCBI Accession Number | Closest species ortholog | Predicted gene function |
| MVA | LJOA01000111 | 72742-76401 | 0.0 | 99 | 69 | [hydroxy methyl glutaryl CoA reductase](https://blast.ncbi.nlm.nih.gov/Blast.cgi#alnHdr_985708128) | OLN85448 | *Colletotrichum chlorophyti* | 3-hydroxy-3-methylglutaryl-CoA reductase |
|  | LJOA01000021 | 245371-246827 | 0.0 | 98 | 81 | acetyl-CoA acetyltransferase | GAP88710 | *Rosellinia necatrix* | Acetoacetyl-CoA thiolase (ERG 10) |
|  | LJOA01000169 | 79713-80841 | 0.0 | 99 | 71 | Farnesyl pyrophosphate synthetase | PMD37588 | *Meliniomyces variabilis* | Farnesyl pyrophosphate synthetase (ERG 20) |
|  | LJOA01000153 | 68268-69534 | 0.0 | 99 | 81 | hydroxymethylglutaryl CoA synthetase | ELA34245 | *Colletotrichum gloeosporioides* | hydroxymethylglutaryl CoA synthetase (ERG 13) |
|  | LJOA01000065 | 15573-16554 | 4e-125 | 98 | 69 | isopentenyl-diphosphate delta-isomerase | OTA63645 | *Hypoxylon sp.* | isopentenyl-diphosphate delta-isomerase |
|  | LJOA01000148 | 403-1696 | 0.0 | 98 | 79 | [mevalonate pyrophosphate decarboxylase](https://blast.ncbi.nlm.nih.gov/Blast.cgi#alnHdr_589109814) | XP_016644508 | *Scedosporium apiospermum* | Diphosphomevalonate decarboxylase |
|  | LJOA01000254 | 95818-97110 | 0.0 | 97 | 73 | Mevalonate kinase | KPA45901 | [*Fusarium*](https://blast.ncbi.nlm.nih.gov/Blast.cgi#alnHdr_685854856) *langsethiae* | Mevalonate kinase (ERG 12) |
|  | LJOA01000327 | 51849-52898 | 3e-151 | 99 | 71 | phosphomevalonate kinase | POR33036 | *Tolypocladium paradoxum* | phosphomevalonate kinase (ERG 8) |
| Sterol | LJOA01000227 | 52821-54418 | 5e-173 | 100 | 65 | 3-keto steroid reductase | ENH81517 | *Colletotrichum orbiculare* | 3-keto sterol reductase (ERG 27) |
|  | LJOA01000225 | 16401-16993 | 2e-169 | 98 | 66 | Ergosterol biosynthesis protein 28 | XP_022474034 | *Colletotrichum orchidophilum* | Ergosterol biosynthesis protein 28 (ERG 28) |
|  | LJOA01000221 | 11019-12656 | 0.0 | 99 | 67 | Delta (14)-sterol reductase | XP_016642425 | *Scedosporium apiospermum* | C-14-sterol reductase (ERG 24) |
|  | LJOA01000165 | 33766-34700 | 3e-186 | 99 | 76 | [fatty acid hydroxylase](https://blast.ncbi.nlm.nih.gov/Blast.cgi#alnHdr_827056543) | XP_016640914 | *Scedosporium apiospermum* | C4 sterol methyl oxidase (ERG 25) |
|  | LJOA01000103 | 16543-18195 | 2e-171 | 77 | 71 | delta24(24(1))-sterol reductase | XM_003050685 | *Nectria haematococca* | C24 sterol reductase (ERG 4) |
|  | LJOA01000253 | 12969-13748 | 2e-156 | 82 | 76 | C5 sterol desaturase | [XM_014096448](https://www.ncbi.nlm.nih.gov/nucleotide/XM_014096448.1?report=genbank&log$=nucltop&blast_rank=1&RID=GUF4EDXZ014) | [*Trichoderma virens*](https://blast.ncbi.nlm.nih.gov/Blast.cgi#alnHdr_927412183) | C5 sterol desaturase (ERG 3) |
|  | LJOA01000070 | 157280-158035 | 1e-162 | 80 | 47 | C8 sterol isomerase | [XM_006961378](https://www.ncbi.nlm.nih.gov/nucleotide/XM_006961378.1?report=genbank&log$=nucltop&blast_rank=1&RID=GUF4EDXZ014) | [*Trichoderma reesei*](https://blast.ncbi.nlm.nih.gov/Blast.cgi#alnHdr_589098834) | C8 sterol isomerase (ERG 2) |
|  | LJOA01000019 | 241473-242924 | 0 | 82 | 75 | Cytochrome P-450 | OLN95588 | *Colletotrichum chlorophyti* | Cytochrome P-450 (ERG 5) |
|  | LJOA01000017 | 7138-7713 | 3e-256 | 75 | 66 | [Delta(24)-sterol C-methyltransferase](https://blast.ncbi.nlm.nih.gov/Blast.cgi#alnHdr_1370882702) | [XM_024468559](https://www.ncbi.nlm.nih.gov/nucleotide/XM_024468559.1?report=genbank&log$=nucltop&blast_rank=1&RID=GUF4EDXZ014) | [*Pseudogymnoascus destructans*](https://blast.ncbi.nlm.nih.gov/Blast.cgi#alnHdr_1370882702) | C24 sterol methyltransferase (ERG 6) |
|  | LJOA01000089 | 182502-184597 | 0.0 | 98 | 70 | Lanosterol synthase | XP_008098265 | *Colletotrichum graminicola* | Lanosterol synthase (ERG 7) |
|  | LJOA01000103 | 41910-43133 | 0.0 | 97 | 61 | Squalene synthase | XP_011395224 | *Neurospora crassa* | Squalene synthase (ERG 9) |
|  | LJOA01000046 | 255443-256770 | 5e-111 | 89 | 75 | proliferating cell nuclear antigen | [XM_008092297.1](https://www.ncbi.nlm.nih.gov/nucleotide/XM_008092297.1?report=genbank&log$=nucltop&blast_rank=1&RID=GUF4EDXZ014) | [*Colletotrichum graminicola*](https://blast.ncbi.nlm.nih.gov/Blast.cgi#alnHdr_827057745) | C14 demethylase (ERG 11) |

| ***C. smalleyi*** | | | | | | | | | |
| --- | --- | --- | --- | --- | --- | --- | --- | --- | --- |
| Pathway | Contig Number | Location | E-value | % Similarity | % Coverage | BLAST sequence description | NCBI Accession Number | Closest species ortholog | Predicted gene function |
| MVA | NETT01000006 | 100146-103778 | 0.0 | 99 | 69 | [hydroxy methyl glutaryl CoA reductase](https://blast.ncbi.nlm.nih.gov/Blast.cgi#alnHdr_985708128) | OLN85448 | *Colletotrichum chlorophyti* | 3-hydroxy-3-methylglutaryl-CoA reductase |
|  | NETT01000081 | 51023-52477 | 0.0 | 99 | 80 | acetyl-CoA acetyltransferase | GAP88710 | *Rosellinia necatrix* | Acetoacetyl-CoA thiolase (ERG 10) |
|  | NETT01000093 | 31713-32922 | 0.0 | 100 | 70 | Farnesyl pyrophosphate synthetase | PMD37588 | *Meliniomyces variabilis* | Farnesyl pyrophosphate synthetase (ERG 20) |
|  | NETT01000763 | 393-2090 | 0.0 | 100 | 81 | hydroxymethylglutaryl CoA synthetase | ELA34245 | *Colletotrichum gloeosporioides* | hydroxymethylglutaryl CoA synthetase (ERG 13) |
|  | NETT01000447 | 15500-16487 | 2e-168 | 99 | 68 | isopentenyl-diphosphate delta-isomerase | OTA63645 | *Hypoxylon sp.* | isopentenyl-diphosphate delta-isomerase |
|  | NETT01000029 | 633-1896 | 0.0 | 98 | 79 | [mevalonate pyrophosphate decarboxylase](https://blast.ncbi.nlm.nih.gov/Blast.cgi#alnHdr_589109814) | XP_016644508 | *Scedosporium apiospermum* | Diphosphomevalonate decarboxylase |
|  | NETT01000653 | 868-2484 | 0.0 | 97 | 73 | Mevalonate kinase | KPA45901 | [*Fusarium*](https://blast.ncbi.nlm.nih.gov/Blast.cgi#alnHdr_685854856) *langsethiae* | Mevalonate kinase (ERG 12) |
|  | NETT01000002 | 10546-11586 | 2e-149 | 98 | 72 | phosphomevalonate kinase | POR33036 | *Tolypocladium paradoxum* | phosphomevalonate kinase (ERG 8) |
|  | NETT01000018 | 18602-19393 | 2e-59 | 97 | 68 | Ergosterol biosynthesis protein 28 | XP_022474034 | *Colletotrichum orchidophilum* | Ergosterol biosynthesis protein 28 (ERG 28) |
| Sterol | NETT01000522 | 12103-13682 | 5e-138 | 100 | 66 | 3-keto steroid reductase | ENH81517 | *Colletotrichum orbiculare* | 3-keto sterol reductase (ERG 27) |
|  | NETT01000193 | 27018-28657 | 0.0 | 99 | 67 | Delta (14)-sterol reductase | XP_016642425 | *Scedosporium apiospermum* | C-14-sterol reductase (ERG 24) |
|  | NETT010000003 | 84687-85801 | 5e-169 | 100 | 75 | [fatty acid hydroxylase](https://blast.ncbi.nlm.nih.gov/Blast.cgi#alnHdr_827056543) | XP_016640914 | *Scedosporium apiospermum* | C4 sterol methyl oxidase (ERG 25) |
|  | NETT01000042 | 15254-16901 | 2e-171 | 77 | 71 | delta24(24(1))-sterol reductase | XM_003050685.1 | *Nectria haematococca* | C24 sterol reductase (ERG 4) |
|  | NETT01000237 | 32124-33306 | 3e-110 | 80 | 77 | C5 sterol desaturase | [XM_014096448.1](https://www.ncbi.nlm.nih.gov/nucleotide/XM_014096448.1?report=genbank&log$=nucltop&blast_rank=1&RID=GUF4EDXZ014) | [*Trichoderma virens*](https://blast.ncbi.nlm.nih.gov/Blast.cgi#alnHdr_927412183) | C5 sterol desaturase (ERG 3) |
|  | NETT01000723 | 8172-8933 | 2e-61 | 80 | 47 | C8 sterol isomerase | [XM_006961378.1](https://www.ncbi.nlm.nih.gov/nucleotide/XM_006961378.1?report=genbank&log$=nucltop&blast_rank=1&RID=GUF4EDXZ014) | [*Trichoderma reesei*](https://blast.ncbi.nlm.nih.gov/Blast.cgi#alnHdr_589098834) | C8 sterol isomerase (ERG 2) |
|  | NETT01000103 | 35777-37728 | 0 | 81 | 75 | Cytochrome P-450 | OLN95588 | *Colletotrichum chlorophyti* | Cytochrome P-450 (ERG 5) |
|  | NETT01000495 | 9231-10540 | 2e-92 | 74 | 68 | [Delta(24)-sterol C-methyltransferase](https://blast.ncbi.nlm.nih.gov/Blast.cgi#alnHdr_1370882702) | [XM_024468559.1](https://www.ncbi.nlm.nih.gov/nucleotide/XM_024468559.1?report=genbank&log$=nucltop&blast_rank=1&RID=GUF4EDXZ014) | [*Pseudogymnoascus destructans*](https://blast.ncbi.nlm.nih.gov/Blast.cgi#alnHdr_1370882702) | C24 sterol methyltransferase (ERG 6) |
|  | NETT01000011 | 25140-27540 | 0.0 | 99 | 69 | Lanosterol synthase | XP_008098265 | *Colletotrichum graminicola* | Lanosterol synthase (ERG 7) |
|  | NETT01000042 | 48087-49786 | 0.0 | 98 | 60 | Squalene synthase | XP_011395224 | *Neurospora crassa* | Squalene synthase (ERG 9) |
|  | NETT01000228 | 25091-26422 | 4e-151 | 88 | 74 | proliferating cell nuclear antigen | [XM_008092297.1](https://www.ncbi.nlm.nih.gov/nucleotide/XM_008092297.1?report=genbank&log$=nucltop&blast_rank=1&RID=GUF4EDXZ014) | [*Colletotrichum graminicola*](https://blast.ncbi.nlm.nih.gov/Blast.cgi#alnHdr_827057745) | C14 demethylase (ERG 11) |

| ***C. harringtonii*** | | | | | | | | | |
| --- | --- | --- | --- | --- | --- | --- | --- | --- | --- |
| Pathway | Contig Number | Location | E-value | % Similarity | % Coverage | BLAST sequence description | NCBI Accession Number | Closest species ortholog | Predicted gene function |
| MVA | MKGM01000007 | 40924-44568 | 0.0 | 99 | 69 | [hydroxy methyl glutaryl CoA reductase](https://blast.ncbi.nlm.nih.gov/Blast.cgi#alnHdr_985708128) | OLN85448 | *Colletotrichum chlorophyti* | 3-hydroxy-3-methylglutaryl-CoA reductase |
|  | MKGM01000206 | 12806-14276 | 0.0 | 98 | 81 | acetyl-CoA acetyltransferase | GAP88710 | *Rosellinia necatrix* | Acetoacetyl-CoA thiolase (ERG 10) |
|  | MKGM01000061 | 92082-93210 | 0.0 | 100 | 70 | Farnesyl pyrophosphate synthetase | PMD37588 | *Meliniomyces variabilis* | Farnesyl pyrophosphate synthetase (ERG 20) |
|  | MKGM01000365 | 11743-13459 | 0.0 | 100 | 81 | hydroxymethylglutaryl CoA synthetase | ELA34245 | *Colletotrichum gloeosporioides* | hydroxymethylglutaryl CoA synthetase (ERG 13) |
|  | MKGM01000215 | 26400-27789 | 3e-153 | 99 | 68 | isopentenyl-diphosphate delta-isomerase | OTA63645 | *Hypoxylon sp.* | isopentenyl-diphosphate delta-isomerase |
|  | MKGM01000025 | 903-2096 | 0.0 | 98 | 79 | [mevalonate pyrophosphate decarboxylase](https://blast.ncbi.nlm.nih.gov/Blast.cgi#alnHdr_589109814) | XP_016644508 | *Scedosporium apiospermum* | Diphosphomevalonate decarboxylase |
|  | MKGM01000006 | 32853-34469 | 0.0 | 97 | 73 | Mevalonate kinase | KPA45901 | [*Fusarium*](https://blast.ncbi.nlm.nih.gov/Blast.cgi#alnHdr_685854856) *langsethiae* | Mevalonate kinase (ERG 12) |
|  | MKGM01000003 | 75993-77033 | 4e-159 | 99 | 71 | phosphomevalonate kinase | POR33036 | *Tolypocladium paradoxum* | phosphomevalonate kinase (ERG 8) |
| Sterol | MKGM01000132 | 52461-54057 | 6e-124 | 98 | 67 | 3-keto steroid reductase | ENH81517 | *Colletotrichum orbiculare* | 3-keto sterol reductase (ERG 27) |
|  | MKGM01000089 | 12602-12278 | 3e-86 | 97 | 68 | Ergosterol biosynthesis protein 28 | XP_022474034 | *Colletotrichum orchidophilum* | Ergosterol biosynthesis protein 28 (ERG 28) |
|  | MKGM01000455 | 1715-2943 | 0.0 | 99 | 67 | Delta (14)-sterol reductase | XP_016642425 | *Scedosporium apiospermum* | C-14-sterol reductase (ERG 24) |
|  | MKGM01000036 | 17304-18420 | 4e-68 | 100 | 75 | [fatty acid hydroxylase](https://blast.ncbi.nlm.nih.gov/Blast.cgi#alnHdr_827056543) | XP_016640914 | *Scedosporium apiospermum* | C4 sterol methyl oxidase (ERG 25) |
|  | MKGM01000302 | 15103-16756 | 3e-94 | 77 | 71 | delta24(24(1))-sterol reductase | XM_003050685.1 | *Nectria haematococca* | C24 sterol reductase (ERG 4) |
|  | MKGM01000014 | 32305-33515 | 2e-156 | 80 | 77 | C5 sterol desaturase | [XM_014096448.1](https://www.ncbi.nlm.nih.gov/nucleotide/XM_014096448.1?report=genbank&log$=nucltop&blast_rank=1&RID=GUF4EDXZ014) | [*Trichoderma virens*](https://blast.ncbi.nlm.nih.gov/Blast.cgi#alnHdr_927412183) | C5 sterol desaturase (ERG 3) |
|  | MKGM01000128 | 38178-38942 | 3e-51 | 80 | 47 | C8 sterol isomerase | [XM_006961378.1](https://www.ncbi.nlm.nih.gov/nucleotide/XM_006961378.1?report=genbank&log$=nucltop&blast_rank=1&RID=GUF4EDXZ014) | [*Trichoderma reesei*](https://blast.ncbi.nlm.nih.gov/Blast.cgi#alnHdr_589098834) | C8 sterol isomerase (ERG 2) |
|  | MKGM01000263 | 398-2333 | 0 | 82 | 75 | Cytochrome P-450 | OLN95588 | *Colletotrichum chlorophyti* | Cytochrome P-450 (ERG 5) |
|  | MKGM01000004 | 211123-212433 | 3e-67 | 74 | 68 | [Delta(24)-sterol C-methyltransferase](https://blast.ncbi.nlm.nih.gov/Blast.cgi#alnHdr_1370882702) | [XM_024468559.1](https://www.ncbi.nlm.nih.gov/nucleotide/XM_024468559.1?report=genbank&log$=nucltop&blast_rank=1&RID=GUF4EDXZ014) | [*Pseudogymnoascus destructans*](https://blast.ncbi.nlm.nih.gov/Blast.cgi#alnHdr_1370882702) | C24 sterol methyltransferase (ERG 6) |
|  | MKGM01000019 | 100742-103150 | 0.0 | 99 | 69 | Lanosterol synthase | XP_008098265 | *Colletotrichum graminicola* | Lanosterol synthase (ERG 7) |
|  | MKGM01000302 | 955-2643 | 0.0 | 98 | 60 | Squalene synthase | XP_011395224 | *Neurospora crassa* | Squalene synthase (ERG 9) |
|  | MKGM01000001 | 276863-278195 | 6e-142 | 88 | 74 | proliferating cell nuclear antigen | [XM_008092297.1](https://www.ncbi.nlm.nih.gov/nucleotide/XM_008092297.1?report=genbank&log$=nucltop&blast_rank=1&RID=GUF4EDXZ014) | [*Colletotrichum graminicola*](https://blast.ncbi.nlm.nih.gov/Blast.cgi#alnHdr_827057745) | C14 demethylase (ERG 11) |

| ***C. albifundus*** | | | | | | | | | |
| --- | --- | --- | --- | --- | --- | --- | --- | --- | --- |
| Pathway | Contig Number | Location | E-value | % Similarity | % Coverage | BLAST sequence description | NCBI Accession Number | Closest species ortholog | Predicted gene function |
| MVA | JSSU01001311 | 30305-33964 | 0.0 | 99 | 69 | [hydroxy methyl glutaryl CoA reductase](https://blast.ncbi.nlm.nih.gov/Blast.cgi#alnHdr_985708128) | OLN85448 | *Colletotrichum chlorophyti* | 3-hydroxy-3-methylglutaryl-CoA reductase |
|  | JSSU01001204 | 29025-30486 | 0.0 | 99 | 80 | acetyl-CoA acetyltransferase | GAP88710 | *Rosellinia necatrix* | Acetoacetyl-CoA thiolase (ERG 10) |
|  | JSSU01001384 | 93224-94355 | 0.0 | 100 | 70 | Farnesyl pyrophosphate synthetase | PMD37588 | *Meliniomyces variabilis* | Farnesyl pyrophosphate synthetase (ERG 20) |
|  | JSSU01001076 | 18306-20371 | 0.0 | 100 | 81 | hydroxymethylglutaryl CoA synthetase | ELA34245 | *Colletotrichum gloeosporioides* | hydroxymethylglutaryl CoA synthetase (ERG 13) |
|  | JSSU01001281 | 32792-33759 | 4e-98 | 100 | 67 | isopentenyl-diphosphate delta-isomerase | OTA63645 | *Hypoxylon sp.* | isopentenyl-diphosphate delta-isomerase |
|  | JSSU01001434 | 662-1739 | 0.0 | 98 | 79 | [mevalonate pyrophosphate decarboxylase](https://blast.ncbi.nlm.nih.gov/Blast.cgi#alnHdr_589109814) | XP_016644508 | *Scedosporium apiospermum* | Diphosphomevalonate decarboxylase |
|  | JSSU01001293 | 49564-51188 | 0.0 | 97 | 73 | Mevalonate kinase | KPA45901 | [*Fusarium*](https://blast.ncbi.nlm.nih.gov/Blast.cgi#alnHdr_685854856) *langsethiae* | Mevalonate kinase (ERG 12) |
|  | JSSU01001404 | 184130-185527 | 3e-151 | 99 | 71 | phosphomevalonate kinase | POR33036 | *Tolypocladium paradoxum* | phosphomevalonate kinase (ERG 8) |
| Sterol | JSSU01001179 | 8943-10431 | 6e-78 | 98 | 68 | 3-keto steroid reductase | ENH81517 | *Colletotrichum orbiculare* | 3-keto sterol reductase (ERG 27) |
|  | JSSU01000295 | 10138-1070 | 6e-79 | 97 | 65 | Ergosterol biosynthesis protein 28 | OLN97423 | *Colletotrichum chlorophyti* | Ergosterol biosynthesis protein 28 (ERG 28) |
|  | JSSU01001012 | 1-1952 | 0.0 | 99 | 67 | Delta (14)-sterol reductase | XP_016642425 | *Scedosporium apiospermum* | C-14-sterol reductase (ERG 24) |
|  | JSSU01001381 | 69961-71029 | 3e-45 | 99 | 76 | [fatty acid hydroxylase](https://blast.ncbi.nlm.nih.gov/Blast.cgi#alnHdr_827056543) | XP_016640914 | *Scedosporium apiospermum* | C4 sterol methyl oxidase (ERG 25) |
|  | JSSU01001386 | 10955-12632 | 2e-165 | 77 | 71 | delta24(24(1))-sterol reductase | XM_003050685.1 | *Nectria haematococca* | C24 sterol reductase (ERG 4) |
|  | JSSU01001250 | 6697-7906 | 2e-98 | 80 | 77 | C5 sterol desaturase | [XM_014096448.1](https://www.ncbi.nlm.nih.gov/nucleotide/XM_014096448.1?report=genbank&log$=nucltop&blast_rank=1&RID=GUF4EDXZ014) | [*Trichoderma virens*](https://blast.ncbi.nlm.nih.gov/Blast.cgi#alnHdr_927412183) | C5 sterol desaturase (ERG 3) |
|  | JSSU01001132 | 26302-27053 | 4e-87 | 82 | 46 | C8 sterol isomerase | [XM_006961378.1](https://www.ncbi.nlm.nih.gov/nucleotide/XM_006961378.1?report=genbank&log$=nucltop&blast_rank=1&RID=GUF4EDXZ014) | [*Trichoderma reesei*](https://blast.ncbi.nlm.nih.gov/Blast.cgi#alnHdr_589098834) | C8 sterol isomerase (ERG 2) |
|  | JSSU01000719 | 1184-3131 | 0 | 82 | 75 | Cytochrome P-450 | OLN95588 | *Colletotrichum chlorophyti* | Cytochrome P-450 (ERG 5) |
|  | JSSU01001056 | 12455-13764 | 3e-167 | 77 | 66 | [Delta(24)-sterol C-methyltransferase](https://blast.ncbi.nlm.nih.gov/Blast.cgi#alnHdr_1370882702) | [XM_024468559.1](https://www.ncbi.nlm.nih.gov/nucleotide/XM_024468559.1?report=genbank&log$=nucltop&blast_rank=1&RID=GUF4EDXZ014) | [*Pseudogymnoascus destructans*](https://blast.ncbi.nlm.nih.gov/Blast.cgi#alnHdr_1370882702) | C24 sterol methyltransferase (ERG 6) |
|  | JSSU01001246 | 2510-4883 | 0.0 | 99 | 69 | Lanosterol synthase | XP_008098265 | *Colletotrichum graminicola* | Lanosterol synthase (ERG 7) |
|  | JSSU01001386 | 73412-75026 | 0.0 | 98 | 60 | Squalene synthase | XP_011395224 | *Neurospora crassa* | Squalene synthase (ERG 9) |
|  | JSSU01001405 | 189620-190954 | 7e-62 | 89 | 75 | proliferating cell nuclear antigen | [XM_008092297.1](https://www.ncbi.nlm.nih.gov/nucleotide/XM_008092297.1?report=genbank&log$=nucltop&blast_rank=1&RID=GUF4EDXZ014) | [*Colletotrichum graminicola*](https://blast.ncbi.nlm.nih.gov/Blast.cgi#alnHdr_827057745) | C14 demethylase (ERG 11) |

| ***C. adiposa*** | | | | | | | | | |
| --- | --- | --- | --- | --- | --- | --- | --- | --- | --- |
| Pathway | Contig Number | Location | E-value | % Similarity | % Coverage | BLAST sequence description | NCBI Accession Number | Closest species ortholog | Predicted gene function |
| MVA | LXGU01000013 | 228537-232175 | 0.0 | 95 | 68 | [hydroxy methyl glutaryl CoA reductase](https://blast.ncbi.nlm.nih.gov/Blast.cgi#alnHdr_985708128) | OLN85448 | *Colletotrichum chlorophyti* | 3-hydroxy-3-methylglutaryl-CoA reductase |
|  | LXGU01000023 | 57486-58959 | 0.0 | 98 | 79 | acetyl-CoA acetyltransferase | KPA40141 | *Fusarium langsethiae* | Acetoacetyl-CoA thiolase (ERG 10) |
|  | LXGU01000044 | 21319-22503 | 9e-179 | 98 | 70 | Farnesyl pyrophosphate synthetase | PNY27092 | *Tolypocladium capitatum* | Farnesyl pyrophosphate synthetase (ERG 20) |
|  | LXGU01000070 | 4201-6052 | 0.0 | 99 | 75 | hydroxymethylglutaryl CoA synthetase | PSR76629 | *Coniella lustricola* | hydroxymethylglutaryl CoA synthetase (ERG 13) |
|  | LXGU01000026 | 25300-26270 | 9e-147 | 100 | 77 | isopentenyl-diphosphate delta-isomerase | KXJ92177 | *Microdochium bolleyi* | isopentenyl-diphosphate delta-isomerase |
|  | LXGU01000004 | 327327-328707 | 0.0 | 80 | 76 | [mevalonate pyrophosphate decarboxylase](https://blast.ncbi.nlm.nih.gov/Blast.cgi#alnHdr_589109814) | XP_016644508 | *Scedosporium apiospermum* | Diphosphomevalonate decarboxylase |
|  | LXGU01000244 | 16499-18093 | 0.0 | 86 | 71 | Mevalonate kinase | XP_023428990 | [*Fusarium fujikuroi*](https://blast.ncbi.nlm.nih.gov/Blast.cgi#alnHdr_685854856) | Mevalonate kinase |
|  | LXGU01000067 | 108771-110143 | 1e-89 | 77 | 82 | phosphomevalonate kinase | XM_018805882.1 | *Trichoderma gamsii* | phosphomevalonate kinase (ERG 8) |
| Sterol | LXGU01000015 | 72807-74270 | 1e-146 | 100 | 47 | Hypothetical | XP_018157938 | *Colletotrichum higginsianum* | 3-keto sterol reductase (ERG 27) |
|  | JSSU01000180 | 39531-40351 | 7e-52 | 97 | 65 | Ergosterol biosynthesis protein 28 | OLN97423 | *Colletotrichum chlorophyti* | Ergosterol biosynthesis protein 28 (ERG 28) |
|  | LXGU01000006 | 2981-4657 | 0.0 | 99 | 68 | Delta (14)-sterol reductase | XP_016642425 | *Scedosporium apiospermum* | C-14-sterol reductase (ERG 24) |
|  | LXGU01000085 | 104392-105356 | 1e-173 | 100 | 78 | [fatty acid hydroxylase](https://blast.ncbi.nlm.nih.gov/Blast.cgi#alnHdr_827056543) | XP_016640914 | *Scedosporium apiospermum* | C4 sterol methyl oxidase (ERG 25) |
|  | LXGU01000022 | 67456-68549 | 2e-177 | 99 | 68 | C5 sterol desaturase | XP_006965542 | *Trichoderma reesei* | C5 sterol desaturase (ERG 3) |
|  | LXGU01000067 | 15786-16652 | 3e-51 | 80 | 57 | C8 sterol isomerase | [XM_006961378.1](https://www.ncbi.nlm.nih.gov/nucleotide/XM_006961378.1?report=genbank&log$=nucltop&blast_rank=1&RID=GUF4EDXZ014) | [*Trichoderma reesei*](https://blast.ncbi.nlm.nih.gov/Blast.cgi#alnHdr_589098834) | C8 sterol isomerase (ERG 2) |
|  | LXGU01000034 | 11982-13566 | 5e-123 | 77 | 51 | [Delta(24(24(1)))-sterol reductase](https://blast.ncbi.nlm.nih.gov/Blast.cgi#alnHdr_629684386) | [XM_007809298.1](https://www.ncbi.nlm.nih.gov/nucleotide/XM_007809298.1?report=genbank&log$=nucltop&blast_rank=1&RID=GUF4EDXZ014) | [*Metarhizium acridum*](https://blast.ncbi.nlm.nih.gov/Blast.cgi#alnHdr_629684386) | C24 sterol reductase (ERG 4) |
|  | LXGU01000005 | 25190-27046 | 0.0 | 99 | 80 | Cytochrome P-450 | PWI70663 | *Purpureocillium lilacinum* | Cytochrome P-450 (ERG 5) |
|  | LXGU01000148 | 5985-7263 | 0.0 | 99 | 84 | [Delta(24)-sterol C-methyltransferase](https://blast.ncbi.nlm.nih.gov/Blast.cgi#alnHdr_1370882702) | KZL83690 | *Colletotrichum incanum* | C24 sterol methyltransferase (ERG 6) |
|  | LXGU01000113 | 19439-24773 | 0.0 | 72 | 49 | Lanosterol synthase | PKS07236 | *Lomentospora prolificans* | Lanosterol synthase (ERG 7) |
|  | LXGU01000034 | 88966-90916 | 0.0 | 98 | 64 | Squalene synthase | XP_011395224 | *Neurospora crassa* | Squalene synthase (ERG 9) |
|  | LXGU01000084 | 40495-42160 | 0.0 | 99 | 71 | C14 demethylase | PKS10573 | *Lomentospora prolificans* | C14 demethylase (ERG 11) |

| ***D.virescens*** |  | | | | | | | | |
| --- | --- | --- | --- | --- | --- | --- | --- | --- | --- |
| Pathway | Contig Number | Location | E-value | % Similarity | % Coverage | BLAST sequence description | NCBI Accession Number | Closest species ortholog | Predicted gene function |
| MVA | LJZU01000036 | 40561-44205 | 0.0 | 90 | 78 | [hydroxy methyl glutaryl CoA reductase](https://blast.ncbi.nlm.nih.gov/Blast.cgi#alnHdr_985708120) | [KU171566.1](https://www.ncbi.nlm.nih.gov/nucleotide/KU171566.1?report=genbank&log$=nucltop&blast_rank=1&RID=GUFVCVUU015) | [*Fusarium poae*](https://blast.ncbi.nlm.nih.gov/Blast.cgi#alnHdr_985708120) | 3-hydroxy-3-methylglutaryl-CoA reductase |
|  | LJZU01000030 | 801-2241 | 0.0 | 99 | 80 | acetyl-CoA C-acetyltransferase | KDN64557 | *Colletotrichum sublineola* | Acetoacetyl-CoA thiolase (ERG 10) |
|  | LJZU01000491 | 30425-31634 | 0.0 | 98 | 72 | [farnesyl pyrophosphate synthetase](https://blast.ncbi.nlm.nih.gov/Blast.cgi#alnHdr_758208525) | CEJ80887 | *Torrubiella hemipterigena* | Farnesyl pyrophosphate synthetase (ERG 20) |
|  | LJZU01000215 | 192267-194739 | 0.0 | 82 | 79 | [hydroxymethylglutaryl-CoA synthase](https://blast.ncbi.nlm.nih.gov/Blast.cgi#alnHdr_1069520645) | ELA34245 | *Colletotrichum gloeosporioides* | hydroxymethylglutaryl CoA synthetase (ERG 13) |
|  | LJZU01000172 | 106281-107291 | 2e-148 | 81 | 77 | isopentenyl-diphosphate delta-isomerase | OTA63645 | *Hypoxylon sp.* | isopentenyl-diphosphate delta-isomerase |
|  | LJZU01000063 | 193210-194476 | 0.0 | 98 | 78 | mevalonate pyrophosphate decarboxylase | XP_016644508 | *Scedosporium apiospermum* | Diphosphomevalonate decarboxylase |
|  | LJZU01000323 | 12388-13990 | 0.0 | 99 | 74 | [mevalonate kinase](https://blast.ncbi.nlm.nih.gov/Blast.cgi#alnHdr_667658612) | KLO96633 | *Fusarium fujikuroi* | Mevalonate kinase (ERG 12) |
|  | LJZU01000332 | 1027-2398 | 5e-157 | 99 | 53 | [phosphomevalonate kinase](https://blast.ncbi.nlm.nih.gov/Blast.cgi#alnHdr_808376609) | POR33036 | *Tolypocladium paradoxum* | phosphomevalonate kinase (ERG 8) |
| Sterol | LJZU01000068 | 210830-212442 | 1e-87 | 79 | 69 | Hypothetical | [XM_003345868.1](https://www.ncbi.nlm.nih.gov/nucleotide/XM_003345868.1?report=genbank&log$=nucltop&blast_rank=1&RID=GUGGXKMA015) | *Sordaria macrospora* | 3-keto sterol reductase (ERG 27) |
|  | LJZU01000225 | 115793-116236 | 3e-71 | 97 | 63 | Ergosterol biosynthesis protein 28 | OLN97423 | *Colletotrichum fioiriani* | Ergosterol biosynthesis protein 28 (ERG 28) |
|  | LJZU01000081 | 108982-110708 | 0.0 | 99 | 68 | Delta (14)-sterol reductase | XP_016642425 | *Scedosporium apiospermum* | C-14-sterol reductase (ERG 24) |
|  | LJZU01000376 | 12394-13582 | 8e-174 | 100 | 75 | [fatty acid hydroxylase](https://blast.ncbi.nlm.nih.gov/Blast.cgi#alnHdr_1248269975) | XP_016640914 | *Scedosporium apiospermum* | C4 sterol methyl oxidase (ERG 25) |
|  | LJZU01000013 | 135592-136736 | 8e-176 | 82 | 74 | C5 sterol desaturase | [XM_006965480.1](https://www.ncbi.nlm.nih.gov/nucleotide/XM_006965480.1?report=genbank&log$=nucltop&blast_rank=1&RID=GUFVCVUU015) | [*Trichoderma reesei*](https://blast.ncbi.nlm.nih.gov/Blast.cgi#alnHdr_589107038) | C5 sterol desaturase (ERG 3) |
|  | LJZU01000264 | 6713-7781 | 5e-93 | 83 | 55 | [C-8 sterol isomerase](https://blast.ncbi.nlm.nih.gov/Blast.cgi#alnHdr_389626796) | [XM_003711004.1](https://www.ncbi.nlm.nih.gov/nucleotide/XM_003711004.1?report=genbank&log$=nucltop&blast_rank=1&RID=GUFVCVUU015) | [*Magnaporthe oryzae*](https://blast.ncbi.nlm.nih.gov/Blast.cgi#alnHdr_389626796) | C8 sterol isomerase (ERG 2) |
|  | LJZU01000239 | 116058-117683 | 0.0 | 85 | 76 | C24 sterol reductase | [XM_003039831.1](https://www.ncbi.nlm.nih.gov/nucleotide/XM_003039831.1?report=genbank&log$=nucltop&blast_rank=1&RID=GUFVCVUU015) | [*Nectria haematococca*](https://blast.ncbi.nlm.nih.gov/Blast.cgi#alnHdr_302881932) | C24 sterol reductase (ERG 4) |
|  | LJZU01000085 | 28819-30706 | 0 | 99 | 78 | Cytochrome P-450 | PWI70663 | *Purpureocillium lilacinum* | Cytochrome P-450 (ERG 5) |
|  | LJZU01000371 | 10876-12178 | 0.0 | 81 | 76 | C24 sterol methyltransferase | [XM_003040984.1](https://www.ncbi.nlm.nih.gov/nucleotide/XM_003040984.1?report=genbank&log$=nucltop&blast_rank=1&RID=GUFVCVUU015) | [*Nectria haematococca*](https://blast.ncbi.nlm.nih.gov/Blast.cgi#alnHdr_302881932) | C24 sterol methyltransferase (ERG 6) |
|  | LJZU01000033 | 180922-183286 | 0.0 | 98 | 71 | Lanosterol synthase | XP_016640229 | *Scedosporium apiospermum* | Lanosterol synthase (ERG 7) |
|  | LJZU01000263 | 49501-51286 | 0.0 | 93 | 67 | Squalene synthase | XP_011395225 | *Neurospora crassa* | Squalene synthase (ERG 9) |
|  | LJZU01000078 | 50756-52420 | 2e-134 | 86 | 74 | 1[4-alpha-demethylase](https://blast.ncbi.nlm.nih.gov/Blast.cgi#alnHdr_1027062207) | [XM_001912614.1](https://www.ncbi.nlm.nih.gov/nucleotide/XM_001912614.1?report=genbank&log$=nucltop&blast_rank=1&RID=GUFVCVUU015) | [*Podospora anserina*](https://blast.ncbi.nlm.nih.gov/Blast.cgi#alnHdr_171695449) | C14 demethylase (ERG 11) |

| ***E. polonica*** | | | | | | | | | |
| --- | --- | --- | --- | --- | --- | --- | --- | --- | --- |
| Pathway | Contig Number | Location | E-value | % Similarity | % Coverage | BLAST sequence description | NCBI Accession Number | Closest species ortholog | Predicted gene function |
| MVA | LXKZ01000181 | 34444-38088 | 0.0 | 95 | 68 | 3-hydroxy-3-methylglutaryl-coenzyme A reductase | OLN85448 | *Colletotrichum chlorophyti* | 3-hydroxy-3-methylglutaryl-CoA reductase |
|  | LXKZ01000050 | 7387-8826 | 0.0 | 99 | 81 | Acetoacetyl-CoA thiolase | KDN64557 | *Colletotrichum sublineola* | Acetoacetyl-CoA thiolase (ERG 10) |
|  | LXKZ01000091 | 19578-20775 | 0.0 | 98 | 70 | farnesyl pyrophosphate synthetase | CEJ80887 | *Torrubiella hemipterigena* | Farnesyl pyrophosphate synthetase (ERG 20) |
|  | LXKZ01000017 | 25123-27008 | 0.0 | 100 | 79 | hydroxymethylglutaryl-CoA synthase | ELA34245 | *Colletotrichum gloeosporioides* | hydroxymethylglutaryl CoA synthetase (ERG 13) |
|  | LXKZ01000294 | 39051-40050 | 2e-146 | 93 | 79 | isopentenyl-diphosphate delta-isomerase | OHX01119 | *Colletotrichum incanum* | isopentenyl-diphosphate delta-isomerase |
|  | LXKZ01000295 | 41258-42507 | 0.0 | 98 | 78 | mevalonate pyrophosphate decarboxylase | XP_016644508 | *Scedosporium apiospermum* | Diphosphomevalonate decarboxylase |
|  | LXKZ01000248 | 15225-16810 | 0.0 | 98 | 75 | Mevalonate kinase | EWZ93722 | *Fusarium oxysporum* | Mevalonate kinase (ERG 12) |
|  | LXKZ01000202 | 82963-84347 | 1e-151 | 99 | 53 | [phosphomevalonate kinase](https://blast.ncbi.nlm.nih.gov/Blast.cgi#alnHdr_808376609) | POR33036 | *Tolypocladium paradoxum* | phosphomevalonate kinase (ERG 8) |
| Sterol | LXKZ01000259 | 33640-35693 | 7e-153 | 84 | 48 | Hypothetical | KXH40884 | *Colletotrichum simmondsii* | 3-keto sterol reductase (ERG 27) |
|  | LXKZ01000223 | 115793-116236 | 3e-69 | 96 | 64 | Ergosterol biosynthesis protein 28 | KZL70202 | *Colletotrichum incanum* | Ergosterol biosynthesis protein 28 (ERG 28) |
|  | LXKZ01000120 | 3434-5141 | 0.0 | 99 | 68 | Delta (14)-sterol reductase | XP_016642425 | *Scedosporium apiospermum* | C-14-sterol reductase (ERG 24) |
|  | LXKZ01000508 | 54671-55834 | 9e-175 | 100 | 75 | C-4 methylsterol oxidase | XP_016640914 | *Scedosporium apiospermum* | C4 sterol methyl oxidase (ERG 25) |
|  | LXKZ01000358 | 40828-41964 | 9e-126 | 67 | 79 | C-5 sterol desaturase | [XM_018809891.1](https://www.ncbi.nlm.nih.gov/nucleotide/XM_018809891.1?report=genbank&log$=nucltop&blast_rank=1&RID=GUGGXKMA015) | *Trichoderma gamsii* | C5 sterol desaturase (ERG 3) |
|  | LXKZ01000594 | 14669-15724 | 1e-114 | 92 | 72 | c-8 sterol isomerase | XP_003649780 | *Thielavia terrestris* | C8 sterol isomerase (ERG 2) |
|  | LXKZ01000432 | 79877-81515 | 0.0 | 86 | 76 | Delta (24(24(1)))-sterol reductase | [XM_009651622.1](https://www.ncbi.nlm.nih.gov/nucleotide/XM_009651622.1?report=genbank&log$=nucltop&blast_rank=1&RID=GUGGXKMA015) | *Verticillium dahliae* | C24 sterol reductase (ERG 4) |
|  | LXKZ01000024 | 85463-87359 | 0.0 | 91 | 76 | Cytochrome P450 | [XM_018317745.1](https://www.ncbi.nlm.nih.gov/nucleotide/XM_018317745.1?report=genbank&log$=nucltop&blast_rank=1&RID=GUGGXKMA015) | *Purpureocillium lilacinum* | Cytochrome P450 (ERG 5) |
|  | LXKZ01000588 | 18728-20031 | 0.0 | 97 | 78 | C24 sterol methyltransferase | [XM_003040984.1](https://www.ncbi.nlm.nih.gov/nucleotide/XM_003040984.1?report=genbank&log$=nucltop&blast_rank=1&RID=GUGGXKMA015) | *Nectria haematococca* | C24 sterol methyltransferase (ERG 6) |
|  | LXKZ01000065 | 68805-71172 | 0.0 | 98 | 71 | Lanosterol synthase | XP_008098265 | *Colletotrichum graminicola* | Lanosterol synthase (ERG 7) |
|  | LXKZ01000419 | 35460-37186 | 0.0 | 90 | 69 | Squalene synthase | XP_011395224 | *Neurospora crassa* | Squalene synthase (ERG 9) |
|  | LXKZ01000291 | 51684-53349 | 0.0 | 98 | 72 | 14-alpha-lanosterol demethylase | PKS10573 | *Lomentospora prolificans* | C14 demethylase (ERG 11) |

| ***E. laricicola*** | | | | | | | | | |
| --- | --- | --- | --- | --- | --- | --- | --- | --- | --- |
| Pathway | Contig Number | Location | E-value | % Similarity | % Coverage | BLAST sequence description | NCBI Accession Number | Closest species ortholog | Predicted gene function |
| MVA | LXGT01000118 | 41765-45298 | 0.0 | 95 | 68 | 3-hydroxy-3-methylglutaryl-coenzyme A reductase | OLN85448 | *Colletotrichum chlorophyti* | 3-hydroxy-3-methylglutaryl-CoA reductase |
|  | LXGT01000007 | 74036-75442 | 0.0 | 99 | 81 | Acetoacetyl-CoA thiolase | KDN64557 | *Colletotrichum sublineola* | Acetoacetyl-CoA thiolase (ERG 10) |
|  | LXGT01000002 | 4523-5710 | 0.0 | 98 | 70 | farnesyl pyrophosphate synthetase | CEJ80887 | *Torrubiella hemipterigena* | Farnesyl pyrophosphate synthetase (ERG 20) |
|  | LXGT01000003 | 25169-27046 | 0.0 | 100 | 79 | hydroxymethylglutaryl-CoA synthase | ELA34245 | *Colletotrichum gloeosporioides* | hydroxymethylglutaryl CoA synthetase (ERG 13) |
|  | LXGT01000614 | 2244-3177 | 3e-69 | 92 | 80 | isopentenyl-diphosphate delta-isomerase | OHX01119 | *Colletotrichum incanum* | isopentenyl-diphosphate delta-isomerase |
|  | LXGT01000251 | 39159-40406 | 0.0 | 98 | 78 | mevalonate pyrophosphate decarboxylase | XP_016644508 | *Scedosporium apiospermum* | Diphosphomevalonate decarboxylase |
|  | LXGT01000169 | 26874-28426 | 0.0 | 98 | 75 | Mevalonate kinase | EWZ93722 | *Fusarium oxysporum* | Mevalonate kinase (ERG 12) |
|  | LXGT01000255 | 15416-16794 | 1e-97 | 99 | 53 | [phosphomevalonate kinase](https://blast.ncbi.nlm.nih.gov/Blast.cgi#alnHdr_808376609) | POR33036 | *Tolypocladium paradoxum* | phosphomevalonate kinase (ERG 8) |
|  | LXGT01000192 | 24984-25564 | 3e-56 | 96 | 64 | Ergosterol biosynthesis protein 28 | KZL70202 | *Colletotrichum incanum* | Ergosterol biosynthesis protein 28 (ERG 28) |
| Sterol | LXGT01000082 | 90423-91848 | 6e-162 | 83 | 49 | Hypothetical | KXH40884 | *Colletotrichum simmondsii* | 3-keto sterol reductase (ERG 27) |
|  | LXGT01000484 | 3414-4956 | 0.0 | 99 | 68 | Delta (14)-sterol reductase | XP_016642425 | *Scedosporium apiospermum* | C-14-sterol reductase (ERG 24) |
|  | LXGT01000483 | 14728-15799 | 6e-164 | 99 | 76 | C-4 methylsterol oxidase | XP_016640914 | *Scedosporium apiospermum* | C4 sterol methyl oxidase (ERG 25) |
|  | LXGT01000121 | 24196-25330 | 7e-138 | 68 | 80 | C-5 sterol desaturase | [XM_018809891.1](https://www.ncbi.nlm.nih.gov/nucleotide/XM_018809891.1?report=genbank&log$=nucltop&blast_rank=1&RID=GUGGXKMA015) | *Trichoderma gamsii* | C5 sterol desaturase (ERG 3) |
|  | LXGT01000017 | 96046-97059 | 2e-153 | 91 | 73 | c-8 sterol isomerase | XP_003649780 | *Thielavia terrestris* | C8 sterol isomerase (ERG 2) |
|  | LXGT01000007 | 115077-116617 | 0.0 | 86 | 76 | Delta (24(24(1)))-sterol reductase | [XM_009651622.1](https://www.ncbi.nlm.nih.gov/nucleotide/XM_009651622.1?report=genbank&log$=nucltop&blast_rank=1&RID=GUGGXKMA015) | *Verticillium dahliae* | C24 sterol reductase (ERG 4) |
|  | LXGT01000133 | 26123-27897 | 0.0 | 91 | 76 | Cytochrome P450 | [XM_018317745.1](https://www.ncbi.nlm.nih.gov/nucleotide/XM_018317745.1?report=genbank&log$=nucltop&blast_rank=1&RID=GUGGXKMA015) | *Purpureocillium lilacinum* | Cytochrome P450 (ERG 5) |
|  | LXGT01000102 | 80377-81675 | 0.0 | 97 | 78 | C24 sterol methyltransferase | [XM_003040984.1](https://www.ncbi.nlm.nih.gov/nucleotide/XM_003040984.1?report=genbank&log$=nucltop&blast_rank=1&RID=GUGGXKMA015) | *Nectria haematococca* | C24 sterol methyltransferase (ERG 6) |
|  | LXGT01000065 | 43700-46031 | 0.0 | 98 | 71 | Lanosterol synthase | XP_008098265 | *Colletotrichum graminicola* | Lanosterol synthase (ERG 7) |
|  | LXGT01000091 | 57815-59469 | 0.0 | 90 | 69 | Squalene synthase | XP_011395224 | *Neurospora crassa* | Squalene synthase (ERG 9) |
|  | LXGT01000167 | 33355-34688 | 0.0 | 98 | 72 | 14-alpha-lanosterol demethylase | PKS10573 | *Lomentospora prolificans* | C14 demethylase (ERG 11) |

| ***H. moniliformis*** | | | | | | | | | |
| --- | --- | --- | --- | --- | --- | --- | --- | --- | --- |
| Pathway | Contig Number | Location | E-value | % Similarity | % Coverage | BLAST sequence description | NCBI Accession Number | Closest species ortholog | Predicted gene function |
| MVA | JMSH01000119 | 7968-11435 | 0.0 | 57 | 79 | hydroxy methyl glutaryl CoA reductase | OLN85448 | *Colletotrichum chlorophyti* | 3-hydroxy-3-methylglutaryl-CoA reductase |
|  | JMSH01000016 | 259503-260888 | 0.0 | 99 | 81 | acetyl-CoA acetyltransferase | KPA40141 | *Fusarium langsethiae* | Acetoacetyl-CoA thiolase (ERG 10) |
|  | JMSH01000013 | 53153-54317 | 0.0 | 97 | 72 | Farnesyl pyrophosphate synthetase | OAA75528 | *Cordyceps confrogosa* | Farnesyl pyrophosphate synthetase (ERG 20) |
|  | JMSH01000008 | 93591-95397 | 0.0 | 99 | 76 | hydroxymethylglutaryl CoA synthetase | CEJ94202 | *Torrubiella hemipterigena* | hydroxymethylglutaryl CoA synthetase (ERG 13) |
|  | JMSH01000004 | 334257-335234 | 1e-148 | 96 | 81 | isopentenyl-diphosphate delta-isomerase | KXJ92177 | *Microdochium bolleyi* | isopentenyl-diphosphate delta-isomerase |
|  | JMSH01000033 | 6749-8057 | 0.0 | 98 | 78 | mevalonate pyrophosphate decarboxylase | PKS12276 | *Lomentospora prolificans* | Diphosphomevalonate decarboxylase |
|  | JMSH01000060 | 67230-68886 | 0.0 | 94 | 72 | [mevalonate kinase](https://blast.ncbi.nlm.nih.gov/Blast.cgi#alnHdr_667658612) | CEI69416 | [*Fusarium*](https://blast.ncbi.nlm.nih.gov/Blast.cgi#alnHdr_685854856) *venenatum* | Mevalonate kinase (ERG 12) |
|  | JMSH01000078 | 45387-46719 | 0.0 | 75 | 87 | [phosphomevalonate kinase](https://blast.ncbi.nlm.nih.gov/Blast.cgi#alnHdr_808376609) | [XM_012337726.1](https://www.ncbi.nlm.nih.gov/nucleotide/XM_012337726.1?report=genbank&log$=nucltop&blast_rank=1&RID=GUFVCVUU015) | [*Pseudozyma hubeiensis*](https://blast.ncbi.nlm.nih.gov/Blast.cgi#alnHdr_808376609) | phosphomevalonate kinase (ERG 8) |
| Sterol | JMSH01000238 | 793628-795079 | 1e-75 | 69 | 79 | Hypothetical | [XM_003345868.1](https://www.ncbi.nlm.nih.gov/nucleotide/XM_003345868.1?report=genbank&log$=nucltop&blast_rank=1&RID=GUGGXKMA015) | *Sordaria macrospora* | 3-keto sterol reductase (ERG 27) |
|  | JMSH01000157 | 24984-25564 | 1e-70 | 97 | 63 | Ergosterol biosynthesis protein 28 | KZL70202 | *Colletotrichum salicis* | Ergosterol biosynthesis protein 28 (ERG 28) |
|  | JMSH01000191 | 8545-9586 | 6e-173 | 100 | 74 | C-4 methylsterol oxidase | XM_016789087.1 | *Scedosporium apiospermum* | C4 sterol methyl oxidase (ERG 25) |
|  | JMSH01000003 | 293729-294829 | 0 | 91 | 81 | C-5 sterol desaturase | XM_014096448.1 | *Trichoderma virens* | C5 sterol desaturase (ERG 3) |
|  | JMSH01000078 | 10801-11650 | 4e-100 | 96 | 63 | C-8 sterol isomerase | XP_018658582 | *Trichoderma gamsii* | C8 sterol isomerase (ERG2) |
|  | JMSH01000144 | 27153-28728 | 2e-171 | 77 | 71 | delta24(24(1))-sterol reductase | XM_003050685.1 | *Nectria haematococca* | C24 sterol reductase (ERG 4) |
|  | JMSH01000014 | 260169-261979 | 0.0 | 86 | 78 | Cytochrome P-450 | XM_003348639.1 | *Sordaria macrospora* | Cytochrome P-450 (ERG 5) |
|  | JMSH01000072 | 101054-102326 | 0.0 | 98 | 82 | sterol 24-C-methyltransferase | XM_018289280.1 | *Pochonia chlamydosporia* | C24 sterol methyltransferase (ERG 6) |
|  | JMSH01000020 | 120433-122754 | 0.0 | 99 | 71 | Lanosterol synthase | XP_008098265 | *Colletotrichum graminicola* | Lanosterol synthase (ERG 7) |
|  | JMSH01000078 | 78491-80233 | 0.0 | 90 | 75 | Squalene synthase | XP_011395224 | *Neurospora crassa* | Squalene synthase (ERG 9) |
|  | JMSH01000078 | 25165-26873 | 0.0 | 99 | 70 | 14-alpha-lanosterol demethylase | PKS10573 | *Lomentospora prolificans* | C14 demethylase (ERG 11) |

| ***H. omanensis*** | | | | | | | | | |
| --- | --- | --- | --- | --- | --- | --- | --- | --- | --- |
| Pathway | Contig Number | Location | E-value | % Similarity | % Coverage | BLAST sequence description | NCBI Accession Number | Closest species ortholog | Predicted gene function |
| MVA | JSUI01006078 | 113107-16571 | 0.0 | 57 | 79 | hydroxy methyl glutaryl CoA reductase | OLN85448 | *Colletotrichum chlorophyti* | 3-hydroxy-3-methylglutaryl-CoA reductase |
|  | JSUI01006535 | 61682-63080 | 0.0 | 99 | 81 | acetyl-CoA acetyltransferase | KPA40141 | *Fusarium langsethiae* | Acetoacetyl-CoA thiolase (ERG 10) |
|  | JSUI01006466 | 35109-36265 | 0.0 | 97 | 72 | Farnesyl pyrophosphate synthetase | OAA75528 | *Cordyceps confrogosa* | Farnesyl pyrophosphate synthetase (ERG 20) |
|  | JSUI01005987 | 6560-8353 | 0.0 | 99 | 76 | hydroxymethylglutaryl CoA synthetase | CEJ94202 | *Torrubiella hemipterigena* | hydroxymethylglutaryl CoA synthetase (ERG 13) |
|  | JSUI01006214 | 11490-12430 | 2e-67 | 97 | 80 | isopentenyl-diphosphate delta-isomerase | KXJ92177 | *Microdochium bolleyi* | isopentenyl-diphosphate delta-isomerase |
|  | JSUI01006238 | 5450-6658 | 0.0 | 98 | 78 | mevalonate pyrophosphate decarboxylase | PKS12276 | *Lomentospora prolificans* | Diphosphomevalonate decarboxylase |
|  | JSUI01006528 | 62290-63943 | 0.0 | 94 | 72 | [mevalonate kinase](https://blast.ncbi.nlm.nih.gov/Blast.cgi#alnHdr_667658612) | CEI69416 | [*Fusarium*](https://blast.ncbi.nlm.nih.gov/Blast.cgi#alnHdr_685854856) *venenatum* | Mevalonate kinase (ERG 12) |
|  | JSUI01006342 | 28952-30280 | 0.0 | 75 | 87 | [phosphomevalonate kinase](https://blast.ncbi.nlm.nih.gov/Blast.cgi#alnHdr_808376609) | [XM_012337726.1](https://www.ncbi.nlm.nih.gov/nucleotide/XM_012337726.1?report=genbank&log$=nucltop&blast_rank=1&RID=GUFVCVUU015) | [*Pseudozyma hubeiensis*](https://blast.ncbi.nlm.nih.gov/Blast.cgi#alnHdr_808376609) | phosphomevalonate kinase (ERG 8) |
| Sterol | JSUI01006145 | 13161-14579 | 1e-68 | 70 | 80 | Hypothetical | [XM_003345868.1](https://www.ncbi.nlm.nih.gov/nucleotide/XM_003345868.1?report=genbank&log$=nucltop&blast_rank=1&RID=GUGGXKMA015) | *Sordaria macrospora* | 3-keto sterol reductase (ERG 27) |
|  | JSUI01006212 | 22584-23164 | 1e-63 | 97 | 63 | Ergosterol biosynthesis protein 28 | KZL70202 | *Colletotrichum salicis* | Ergosterol biosynthesis protein 28 (ERG 28) |
|  | JSUI01006352 | 9128-10255 | 5e-96 | 99 | 75 | C-4 methylsterol oxidase | XM_016789087.1 | *Scedosporium apiospermum* | C4 sterol methyl oxidase (ERG 25) |
|  | JSUI01006513 | 7119-8205 | 0 | 91 | 81 | C-5 sterol desaturase | XM_014096448.1 | *Trichoderma virens* | C5 sterol desaturase (ERG 3) |
|  | JSUI01006342 | 1815-2610 | 3e-69 | 97 | 62 | C-8 sterol isomerase | XP_018658582 | *Trichoderma gamsii* | C8 sterol isomerase (ERG2) |
|  | JSUI01006211 | 20675-22202 | 1e-125 | 76 | 70 | delta24(24(1))-sterol reductase | XM_003050685.1 | *Nectria haematococca* | C24 sterol reductase (ERG 4) |
|  | JSUI01006408 | 1410-3217 | 0.0 | 86 | 78 | Cytochrome P-450 | XM_003348639.1 | *Sordaria macrospora* | Cytochrome P-450 (ERG 5) |
|  | JSUI01006380 | 4775-6044 | 0.0 | 98 | 82 | sterol 24-C-methyltransferase | XM_018289280.1 | *Pochonia chlamydosporia* | C24 sterol methyltransferase (ERG 6) |
|  | JSUI01006539 | 34303-36356 | 0.0 | 99 | 71 | Lanosterol synthase | XP_008098265 | *Colletotrichum graminicola* | Lanosterol synthase (ERG 7) |
|  | JSUI01006342 | 14838-16503 | 0.0 | 90 | 75 | Squalene synthase | XP_011395224 | *Neurospora crassa* | Squalene synthase (ERG 9) |
|  | JSUI01006342 | 18637-20009 | 0.0 | 99 | 70 | 14-alpha-lanosterol demethylase | PKS10573 | *Lomentospora prolificans* | C14 demethylase (ERG 11) |

| ***H. savannae*** | | | | | | | | | |
| --- | --- | --- | --- | --- | --- | --- | --- | --- | --- |
| Pathway | Contig Number | Location | E-value | % Similarity | % Coverage | BLAST sequence description | NCBI Accession Number | Closest species ortholog | Predicted gene function |
| MVA | JCZG01000133 | 7954-11505 | 0.0 | 57 | 79 | hydroxy methyl glutaryl CoA reductase | OLN85448 | *Colletotrichum chlorophyti* | 3-hydroxy-3-methylglutaryl-CoA reductase |
|  | JCZG01000014 | 304307-305666 | 0.0 | 99 | 81 | acetyl-CoA acetyltransferase | KPA40141 | *Fusarium langsethiae* | Acetoacetyl-CoA thiolase (ERG 10) |
|  | JCZG01000024 | 29108-30028 | 0.0 | 97 | 72 | Farnesyl pyrophosphate synthetase | OAA75528 | *Cordyceps confrogosa* | Farnesyl pyrophosphate synthetase (ERG 20) |
|  | JCZG01000005 | 69591-71106 | 0.0 | 99 | 76 | hydroxymethylglutaryl CoA synthetase | CEJ94202 | *Torrubiella hemipterigena* | hydroxymethylglutaryl CoA synthetase (ERG 13) |
|  | JCZG01000021 | 89743-90686 | 1e-112 | 96 | 81 | isopentenyl-diphosphate delta-isomerase | KXJ92177 | *Microdochium bolleyi* | isopentenyl-diphosphate delta-isomerase |
|  | JCZG01000005 | 11125-12984 | 0.0 | 98 | 78 | mevalonate pyrophosphate decarboxylase | PKS12276 | *Lomentospora prolificans* | Diphosphomevalonate decarboxylase |
|  | JCZG01000004 | 119424-120716 | 0.0 | 94 | 72 | [mevalonate kinase](https://blast.ncbi.nlm.nih.gov/Blast.cgi#alnHdr_667658612) | CEI69416 | [*Fusarium*](https://blast.ncbi.nlm.nih.gov/Blast.cgi#alnHdr_685854856) *venenatum* | Mevalonate kinase (ERG 12) |
|  | JCZG01000001 | 198460-199779 | 0.0 | 75 | 87 | [phosphomevalonate kinase](https://blast.ncbi.nlm.nih.gov/Blast.cgi#alnHdr_808376609) | [XM_012337726.1](https://www.ncbi.nlm.nih.gov/nucleotide/XM_012337726.1?report=genbank&log$=nucltop&blast_rank=1&RID=GUFVCVUU015) | [*Pseudozyma hubeiensis*](https://blast.ncbi.nlm.nih.gov/Blast.cgi#alnHdr_808376609) | phosphomevalonate kinase (ERG 8) |
| Sterol | JCZG01000044 | 124952-126370 | 2e-127 | 68 | 77 | Hypothetical | [XM_003345868.1](https://www.ncbi.nlm.nih.gov/nucleotide/XM_003345868.1?report=genbank&log$=nucltop&blast_rank=1&RID=GUGGXKMA015) | *Sordaria macrospora* | 3-keto sterol reductase (ERG 27) |
|  | JCZG01000067 | 18623-19246 | 1e-65 | 97 | 63 | Ergosterol biosynthesis protein 28 | KZL70202 | *Colletotrichum salicis* | Ergosterol biosynthesis protein 28 (ERG 28) |
|  | JCZG01000001 | 65283-66485 | 0.0 | 99 | 68 | Delta (14)-sterol reductase | XP_016642425 | *Scedosporium apiospermum* | C-14-sterol reductase (ERG 24) |
|  | JCZG01000134 | 20144-21070 | 5e-139 | 99 | 76 | C-4 methylsterol oxidase | XM_016789087.1 | *Scedosporium apiospermum* | C4 sterol methyl oxidase (ERG 25) |
|  | JCZG01000048 | 111663-112749 | 0 | 91 | 81 | C-5 sterol desaturase | XM_014096448.1 | *Trichoderma virens* | C5 sterol desaturase (ERG 3) |
|  | JCZG01000001 | 55007-55800 | 3e-89 | 96 | 63 | C-8 sterol isomerase | XP_018658582 | *Trichoderma gamsii* | C8 sterol isomerase (ERG2) |
|  | JCZG01000155 | 8033-8938 | 1e-86 | 75 | 72 | delta24(24(1))-sterol reductase | XM_003050685.1 | *Nectria haematococca* | C24 sterol reductase (ERG 4) |
|  | JCZG01000068 | 118135-119941 | 0.0 | 86 | 78 | Cytochrome P-450 | XM_003348639.1 | *Sordaria macrospora* | Cytochrome P-450 (ERG 5) |
|  | JCZG01000026 | 44619-45887 | 0.0 | 98 | 82 | sterol 24-C-methyltransferase | XM_018289280.1 | *Pochonia chlamydosporia* | C24 sterol methyltransferase (ERG 6) |
|  | JCZG01000007 | 240904-243222 | 0.0 | 99 | 71 | Lanosterol synthase | XP_008098265 | *Colletotrichum graminicola* | Lanosterol synthase (ERG 7) |
|  | JCZG01000001 | 346899-348566 | 0.0 | 90 | 75 | Squalene synthase | XP_011395224 | *Neurospora crassa* | Squalene synthase (ERG 9) |
|  | JCZG01000001 | 285466-286838 | 0.0 | 99 | 70 | 14-alpha-lanosterol demethylase | PKS10573 | *Lomentospora prolificans* | C14 demethylase (ERG 11) |

| ***H. bhutanensis*** | | | | | | | | | |
| --- | --- | --- | --- | --- | --- | --- | --- | --- | --- |
| Pathway | Contig Number | Location | E-value | % Similarity | % Coverage | BLAST sequence description | NCBI Accession Number | Closest species ortholog | Predicted gene function |
| MVA | MJMS01000113 | 57338-60768 | 0.0 | 57 | 79 | hydroxy methyl glutaryl CoA reductase | OLN85448 | *Colletotrichum chlorophyti* | 3-hydroxy-3-methylglutaryl-CoA reductase |
|  | MJMS01000060 | 55717-56724 | 0.0 | 99 | 81 | acetyl-CoA acetyltransferase | KPA40141 | *Fusarium langsethiae* | Acetoacetyl-CoA thiolase (ERG 10) |
|  | MJMS01000004 | 232193-233346 | 0.0 | 97 | 72 | Farnesyl pyrophosphate synthetase | OAA75528 | *Cordyceps confrogosa* | Farnesyl pyrophosphate synthetase (ERG 20) |
|  | MJMS01000043 | 189648-191441 | 0.0 | 99 | 76 | hydroxymethylglutaryl CoA synthetase | CEJ94202 | *Torrubiella hemipterigena* | hydroxymethylglutaryl CoA synthetase (ERG 13) |
|  | MJMS01000001 | 142914-143854 | 2e-84 | 95 | 83 | isopentenyl-diphosphate delta-isomerase | KXJ92177 | *Microdochium bolleyi* | isopentenyl-diphosphate delta-isomerase |
|  | MJMS01000032 | 7769-9069 | 0.0 | 98 | 78 | mevalonate pyrophosphate decarboxylase | PKS12276 | *Lomentospora prolificans* | Diphosphomevalonate decarboxylase |
|  | MJMS01000014 | 314128-315772 | 0.0 | 94 | 72 | [mevalonate kinase](https://blast.ncbi.nlm.nih.gov/Blast.cgi#alnHdr_667658612) | CEI69416 | [*Fusarium*](https://blast.ncbi.nlm.nih.gov/Blast.cgi#alnHdr_685854856) *venenatum* | Mevalonate kinase (ERG 12) |
|  | MJMS01000021 | 66733-68049 | 0.0 | 75 | 87 | [phosphomevalonate kinase](https://blast.ncbi.nlm.nih.gov/Blast.cgi#alnHdr_808376609) | [XM_012337726.1](https://www.ncbi.nlm.nih.gov/nucleotide/XM_012337726.1?report=genbank&log$=nucltop&blast_rank=1&RID=GUFVCVUU015) | [*Pseudozyma hubeiensis*](https://blast.ncbi.nlm.nih.gov/Blast.cgi#alnHdr_808376609) | phosphomevalonate kinase (ERG 8) |
|  | MJMS01000123 | 4372-4952 | 1e-64 | 97 | 63 | Ergosterol biosynthesis protein 28 | KZL70202 | *Colletotrichum salicis* | Ergosterol biosynthesis protein 28 (ERG 28) |
| Sterol | MJMS01000028 | 115730-117148 | 1e-29 | 68 | 81 | Hypothetical | [XM_003345868.1](https://www.ncbi.nlm.nih.gov/nucleotide/XM_003345868.1?report=genbank&log$=nucltop&blast_rank=1&RID=GUGGXKMA015) | *Sordaria macrospora* | 3-keto sterol reductase (ERG 27) |
|  | MJMS01000021 | 136970-138172 | 0.0 | 99 | 68 | Delta (14)-sterol reductase | XP_016642425 | *Scedosporium apiospermum* | C-14-sterol reductase (ERG 24) |
|  | MJMS01000029 | 53213-54139 | 3e-186 | 100 | 75 | C-4 methylsterol oxidase | XM_016789087.1 | *Scedosporium apiospermum* | C4 sterol methyl oxidase (ERG 25) |
|  | MJMS01000036 | 109823-110596 | 0 | 91 | 81 | C-5 sterol desaturase | XM_014096448.1 | *Trichoderma virens* | C5 sterol desaturase (ERG 3) |
|  | MJMS01000021 | 255011-255804 | 3e-96 | 97 | 62 | C-8 sterol isomerase | XP_018658582 | *Trichoderma gamsii* | C8 sterol isomerase (ERG2) |
|  | MJMS01000062 | 97763-99290 | 1e-128 | 76 | 73 | delta24(24(1))-sterol reductase | XM_003050685.1 | *Nectria haematococca* | C24 sterol reductase (ERG 4) |
|  | MJMS01000009 | 67720-69524 | 0.0 | 86 | 78 | Cytochrome P-450 | XM_003348639.1 | *Sordaria macrospora* | Cytochrome P-450 (ERG 5) |
|  | MJMS01000035 | 206031-207307 | 0.0 | 98 | 82 | sterol 24-C-methyltransferase | XM_018289280.1 | *Pochonia chlamydosporia* | C24 sterol methyltransferase (ERG 6) |
|  | MJMS01000017 | 252221-254539 | 0.0 | 99 | 71 | Lanosterol synthase | XP_008098265 | *Colletotrichum graminicola* | Lanosterol synthase (ERG 7) |
|  | MJMS01000021 | 12555-14221 | 0.0 | 90 | 75 | Squalene synthase | XP_011395224 | *Neurospora crassa* | Squalene synthase (ERG 9) |
|  | MJMS01000021 | 44829-46201 | 0.0 | 99 | 70 | 14-alpha-lanosterol demethylase | PKS10573 | *Lomentospora prolificans* | C14 demethylase (ERG 11) |

| ***H. decipiens*** | | | | | | | | | |
| --- | --- | --- | --- | --- | --- | --- | --- | --- | --- |
| Pathway | Contig Number | Location | E-value | % Similarity | % Coverage | BLAST sequence description | NCBI Accession Number | Closest species ortholog | Predicted gene function |
| MVA | NETU00000165 | 35883-39434 | 0.0 | 57 | 79 | hydroxy methyl glutaryl CoA reductase | OLN85448 | *Colletotrichum chlorophyti* | 3-hydroxy-3-methylglutaryl-CoA reductase |
|  | NETU00000110 | 165638-166997 | 0.0 | 99 | 81 | acetyl-CoA acetyltransferase | KPA40141 | *Fusarium langsethiae* | Acetoacetyl-CoA thiolase (ERG 10) |
|  | NETU00000022 | 38480-39636 | 0.0 | 97 | 72 | Farnesyl pyrophosphate synthetase | OAA75528 | *Cordyceps confrogosa* | Farnesyl pyrophosphate synthetase (ERG 20) |
|  | NETU00000142 | 88930-90723 | 0.0 | 99 | 76 | hydroxymethylglutaryl CoA synthetase | CEJ94202 | *Torrubiella hemipterigena* | hydroxymethylglutaryl CoA synthetase (ERG 13) |
|  | NETU00000174 | 83099-84039 | 1e-165 | 95 | 83 | isopentenyl-diphosphate delta-isomerase | KXJ92177 | *Microdochium bolleyi* | isopentenyl-diphosphate delta-isomerase |
|  | NETU00000065 | 11759-13066 | 0.0 | 98 | 78 | mevalonate pyrophosphate decarboxylase | PKS12276 | *Lomentospora prolificans* | Diphosphomevalonate decarboxylase |
|  | NETU00000222 | 29109-30752 | 0.0 | 94 | 72 | [mevalonate kinase](https://blast.ncbi.nlm.nih.gov/Blast.cgi#alnHdr_667658612) | CEI69416 | [*Fusarium*](https://blast.ncbi.nlm.nih.gov/Blast.cgi#alnHdr_685854856) *venenatum* | Mevalonate kinase (ERG 12) |
|  | NETU00000079 | 27903-29220 | 0.0 | 75 | 87 | [phosphomevalonate kinase](https://blast.ncbi.nlm.nih.gov/Blast.cgi#alnHdr_808376609) | [XM_012337726](https://www.ncbi.nlm.nih.gov/nucleotide/XM_012337726.1?report=genbank&log$=nucltop&blast_rank=1&RID=GUFVCVUU015) | [*Pseudozyma hubeiensis*](https://blast.ncbi.nlm.nih.gov/Blast.cgi#alnHdr_808376609) | phosphomevalonate kinase (ERG 8) |
| Sterol | NETU00000238 | 60008-61426 | 1e-69 | 70 | 79 | Hypothetical | [XM_003345868](https://www.ncbi.nlm.nih.gov/nucleotide/XM_003345868.1?report=genbank&log$=nucltop&blast_rank=1&RID=GUGGXKMA015) | *Sordaria macrospora* | 3-keto sterol reductase (ERG 27) |
|  | NETU00000217 | 24161-24739 | 1e-64 | 97 | 63 | Ergosterol biosynthesis protein 28 | KZL70202 | *Colletotrichum salicis* | Ergosterol biosynthesis protein 28 (ERG 28) |
|  | NETU00000002 | 38110-39036 | 5e-163 | 100 | 75 | C-4 methylsterol oxidase | XM_016789087 | *Scedosporium apiospermum* | C4 sterol methyl oxidase (ERG 25) |
|  | NETU00000275 | 58741-59840 | 0 | 91 | 81 | C-5 sterol desaturase | XM_014096448 | *Trichoderma virens* | C5 sterol desaturase (ERG 3) |
|  | NETU00000079 | 126728-127521 | 2e-89 | 96 | 63 | C-8 sterol isomerase | XP_018658582 | *Trichoderma gamsii* | C8 sterol isomerase (ERG2) |
|  | NETU00000171 | 25350-26790 | 2e-169 | 77 | 71 | delta24(24(1))-sterol reductase | XM_003050685.1 | *Nectria haematococca* | C24 sterol reductase (ERG 4) |
|  | NETU00000079 | 116684-118491 | 0.0 | 86 | 78 | Cytochrome P-450 | XM_003348639.1 | *Sordaria macrospora* | Cytochrome P-450 (ERG 5) |
|  | NETU00000208 | 13261-14529 | 0.0 | 98 | 82 | sterol 24-C-methyltransferase | XM_018289280.1 | *Pochonia chlamydosporia* | C24 sterol methyltransferase (ERG 6) |
|  | NETU00000216 | 75930-78248 | 0.0 | 99 | 71 | Lanosterol synthase | XP_008098265 | *Colletotrichum graminicola* | Lanosterol synthase (ERG 7) |
|  | NETU00000037 | 78482-80144 | 0.0 | 90 | 75 | Squalene synthase | XP_011395224 | *Neurospora crassa* | Squalene synthase (ERG 9) |
|  | NETU00000037 | 17203-18575 | 0.0 | 99 | 70 | 14-alpha-lanosterol demethylase | PKS10573 | *Lomentospora prolificans* | C14 demethylase (ERG 11) |

| ***T.punctulata*** |  | | | | | | | | |
| --- | --- | --- | --- | --- | --- | --- | --- | --- | --- |
| Pathway | Contig Number | Location | E-value | % Similarity | % Coverage | BLAST sequence description | NCBI Accession Number | Closest species ortholog | Predicted gene function |
| MVA | LAEV01002104 | 23774-27259 | 0.0 | 90 | 77 | 3-hydroxy-3-methylglutaryl coenzyme A reductase | XM_018300392.1 | *Colletotrichum higginsianum* | 3-hydroxy-3-methylglutaryl-CoA reductase |
|  | LAEV01002237 | 248092-249487 | 0.0 | 99 | 81 | acetyl-CoA C-acetyltransferase | XP_018241466 | *Fusarium oxysporum* | Acetoacetyl-CoA thiolase (ERG 10) |
|  | LAEV01001500 | 20155-21358 | 0.0 | 98 | 73 | farnesyl-pyrophosphate synthetase | XP_016639693 | *Scedosporium apiospermum* | Farnesyl pyrophosphate synthetase (ERG 20) |
|  | LAEV01001101 | 22903-24700 | 0.0 | 100 | 81 | hydroxymethylglutaryl-CoA synthase | ELA34245 | *Colletotrichum gloeosporioides* | hydroxymethylglutaryl CoA synthetase (ERG 13) |
|  | LAEV01000658 | 25490-26531 | 3e-152 | 100 | 80 | isopentenyl-diphosphate delta-isomerase | OTA63645 | *Hypoxylon sp.* | isopentenyl-diphosphate delta-isomerase |
|  | LAEV01000758 | 116285-117530 | 0.0 | 98 | 80 | mevalonate pyrophosphate decarboxylase | XP_016644508 | *Scedosporium apiospermum* | Diphosphomevalonate decarboxylase |
|  | LAEV01001554 | 67614-69195 | 0.0 | 92 | 77 | Mevalonate kinase | XP_018748980 | *Fusarium verticillioides* | Mevalonate kinase (ERG 12) |
|  | LAEV01002291 | 71033-72491 | 2e-178 | 99 | 59 | phosphomevalonate kinase | XP_003712127 | *Magnaporthe oryzae* | phosphomevalonate kinase (ERG 8) |
| Sterol | LAEV01001792 | 15451-17027 | 1e-142 | 99 | 48 | Hypothetical | [XM_003345868.1](https://www.ncbi.nlm.nih.gov/nucleotide/XM_003345868.1?report=genbank&log$=nucltop&blast_rank=1&RID=GUGGXKMA015) | *Colletotrichum nymphaeae* | 3-keto sterol reductase (ERG 27) |
|  | LAEV01001608 | 68012-68666 | 1e-89 | 97 | 63 | Ergosterol biosynthesis protein 28 | KZL70202 | *Colletotrichum salicis* | Ergosterol biosynthesis protein 28 (ERG 28) |
|  | LAEV01001971 | 118356-119978 | 0.0 | 99 | 68 | delta14-sterol reductase | XP_016642425 | *Scedosporium apiospermum* | C-14-sterol reductase (ERG 24) |
|  | LAEV01001610 | 72629-73866 | 7e-175 | 100 | 75 | Fatty acid hydroxylase | XP_016640914 | *Scedosporium apiospermum* | C4 sterol methyl oxidase (ERG 25) |
|  | LAEV01001877 | 87002-88104 | 1e-178 | 75 | 82 | C-5 sterol desaturase | XM_006965480.1 | *Trichoderma reesei* | C5 sterol desaturase (ERG 3) |
|  | LAEV01001305 | 2421-3275 | 6e-116 | 100 | 69 | C-8 sterol isomerase | XP_003649780 | *Thielavia terrestris* | C8 sterol isomerase (ERG2) |
|  | LAEV01002192 | 285290-287015 | 1e-124 | 99 | 80 | Delta (24(24(1)))-sterol reductase | XM_009651622.1 | *Verticillium dahliae* | C24 sterol reductase (ERG 4) |
|  | LAEV01001896 | 98682-100559 | 0.0 | 84 | 77 | Cytochrome P450 | XM_003348639.1 | *Sordaria macrospora* | Cytochrome P450 (ERG 5) |
|  | LAEV01000101 | 14001-15304 | 0.0 | 97 | 83 | sterol 24-C-methyltransferase | XM_014085521.1 | *Trichoderma atroviride* | C24 sterol methyltransferase (ERG 6) |
|  | LAEV01002059 | 47857-50215 | 0.0 | 98 | 72 | Lanosterol synthase | XP_016640229 | *Scedosporium apiospermum* | Lanosterol synthase (ERG 7) |
|  | LAEV01000658 | 148305-150005 | 0.0 | 90 | 79 | Squalene synthase | XP_011395224 | *Neurospora crassa* | Squalene synthase (ERG 9) |
|  | LAEV01002193 | 47542-49213 | 0.0 | 99 | 74 | eburicol 14a-demethylase | PKS10573 | *Lomentospora prolificans* | C14 demethylase (ERG 11) |

| ***T. musarum*** | | | | | | | | | |
| --- | --- | --- | --- | --- | --- | --- | --- | --- | --- |
| Pathway | Contig Number | Location | E-value | % Similarity | % Coverage | BLAST sequence description | NCBI Accession Number | Closest species ortholog | Predicted gene function |
| MVA | LKBB01000064 | 81813-85367 | 0.0 | 90 | 77 | 3-hydroxy-3-methylglutaryl coenzyme A reductase | XM_018300392.1 | *Colletotrichum higginsianum* | 3-hydroxy-3-methylglutaryl-CoA reductase |
|  | LKBB01000019 | 31663-33046 | 0.0 | 99 | 81 | acetyl-CoA C-acetyltransferase | XP_018241466 | *Fusarium oxysporum* | Acetoacetyl-CoA thiolase (ERG 10) |
|  | LKBB01000344 | 3979-5172 | 0.0 | 98 | 73 | farnesyl-pyrophosphate synthetase | XP_016639693 | *Scedosporium apiospermum* | Farnesyl pyrophosphate synthetase (ERG 20) |
|  | LKBB01000070 | 24794-26616 | 0.0 | 100 | 81 | hydroxymethylglutaryl-CoA synthase | ELA34245 | *Colletotrichum gloeosporioides* | hydroxymethylglutaryl CoA synthetase (ERG 13) |
|  | LKBB01000023 | 60474-61471 | 2e-94 | 100 | 80 | isopentenyl-diphosphate delta-isomerase | OTA63645 | *Hypoxylon sp.* | isopentenyl-diphosphate delta-isomerase |
|  | LKBB01000058 | 26285-27530 | 0.0 | 98 | 80 | mevalonate pyrophosphate decarboxylase | XP_016644508 | *Scedosporium apiospermum* | Diphosphomevalonate decarboxylase |
|  | LKBB01000094 | 55526-57110 | 0.0 | 92 | 77 | Mevalonate kinase | XP_018748980 | *Fusarium verticillioides* | Mevalonate kinase (ERG 12) |
|  | LKBB01000084 | 53785-55148 | 1e-157 | 99 | 59 | phosphomevalonate kinase | XP_003712127 | *Magnaporthe oryzae* | phosphomevalonate kinase (ERG 8) |
| Sterol | LKBB01000058 | 16034-17487 | 1e-98 | 99 | 48 | Hypothetical | [XM_003345868.1](https://www.ncbi.nlm.nih.gov/nucleotide/XM_003345868.1?report=genbank&log$=nucltop&blast_rank=1&RID=GUGGXKMA015) | *Colletotrichum nymphaeae* | 3-keto sterol reductase (ERG 27) |
|  | LKBB01000225 | 32644-33209 | 1e-54 | 97 | 63 | Ergosterol biosynthesis protein 28 | KZL70202 | *Colletotrichum salicis* | Ergosterol biosynthesis protein 28 (ERG 28) |
|  | LKBB01000014 | 17356-18978 | 0.0 | 99 | 68 | delta14-sterol reductase | XP_016642425 | *Scedosporium apiospermum* | C-14-sterol reductase (ERG 24) |
|  | LKBB01000238 | 29322-30373 | 6e-41 | 100 | 75 | Fatty acid hydroxylase | XP_016640914 | *Scedosporium apiospermum* | C4 sterol methyl oxidase (ERG 25) |
|  | LKBB01000006 | 233144-234235 | 1e-54 | 76 | 81 | C-5 sterol desaturase | XM_006965480.1 | *Trichoderma reesei* | C5 sterol desaturase (ERG 3) |
|  | LKBB01000326 | 1736-2542 | 5e-54 | 100 | 70 | C-8 sterol isomerase | XP_003649780 | *Thielavia terrestris* | C8 sterol isomerase (ERG2) |
|  | LKBB01000480 | 159-1585 | 1e-112 | 100 | 81 | Delta (24(24(1)))-sterol reductase | XM_009651622.1 | *Verticillium dahliae* | C24 sterol reductase (ERG 4) |
|  | LKBB01000216 | 36550-38344 | 0.0 | 84 | 77 | Cytochrome P450 | XM_003348639.1 | *Sordaria macrospora* | Cytochrome P450 (ERG 5) |
|  | LKBB01000220 | 7537-8853 | 0.0 | 97 | 83 | sterol 24-C-methyltransferase | XM_014085521.1 | *Trichoderma atroviride* | C24 sterol methyltransferase (ERG 6) |
|  | LKBB01000079 | 107610-109674 | 0.0 | 98 | 72 | Lanosterol synthase | XP_016640229 | *Scedosporium apiospermum* | Lanosterol synthase (ERG 7) |
|  | LKBB01000023 | 182214-183761 | 0.0 | 90 | 79 | Squalene synthase | XP_011395224 | *Neurospora crassa* | Squalene synthase (ERG 9) |
|  | LKBB01000134 | 46892-48227 | 0.0 | 99 | 74 | eburicol 14a-demethylase | PKS10573 | *Lomentospora prolificans* | C14 demethylase (ERG 11) |

| ***Be. basicola*** | | | | | | | | | |
| --- | --- | --- | --- | --- | --- | --- | --- | --- | --- |
| Pathway | Contig Number | Location | E-value | % Similarity | % Coverage | BLAST sequence description | NCBI Accession Number | Closest species ortholog | Predicted gene function |
| MVA | 7 | 2007451-2010933 | 0.0 | 99 | 69 | 3-hydroxy-3-methylglutaryl-coenzyme A reductase | OLN85448 | *Colletotrichum chlorophyti* | 3-hydroxy-3-methylglutaryl-CoA reductase |
|  | 1 | 143678-145114 | 0.0 | 99 | 80 | Acetoacetyl-CoA thiolase | GAP88710 | *Rosellinia necatrix* | Acetoacetyl-CoA thiolase (ERG 10) |
|  | 23 | 151534-152695 | 0.0 | 100 | 74 | farnesyl pyrophosphate synthetase | [XP_003050078](https://www.ncbi.nlm.nih.gov/nucleotide/JQ780841.1?report=genbank&log$=nucltop&blast_rank=1&RID=GUGGXKMA015) | *Nectria haematococca* | Farnesyl pyrophosphate synthetase (ERG 20) |
|  | 4 | 356055-357710 | 0.0 | 100 | 79 | hydroxymethylglutaryl-CoA synthase | ELA34245 | *Colletotrichum gloeosporioides* | hydroxymethylglutaryl CoA synthetase (ERG 13) |
|  | 12 | 662622-663612 | 4e-122 | 97 | 66 | isopentenyl-diphosphate delta-isomerase | OTA63645 | *Hypoxylon sp.* | isopentenyl-diphosphate delta-isomerase |
|  | 2 | 554185-555464 | 0.0 | 99 | 80 | mevalonate pyrophosphate decarboxylase | PKS12276 | *Lomentospora prolificans* | Diphosphomevalonate decarboxylase |
|  | 31 | 208264-209845 | 0.0 | 69 | 71 | mevalonate-3-phosphate-5-kinase | KPA45901 | [*Fusarium*](https://blast.ncbi.nlm.nih.gov/Blast.cgi#alnHdr_685854856) *langsethiae* | Mevalonate kinase (ERG 12) |
|  | 15 | 379107-380538 | 2e-175 | 99 | 58 | [phosphomevalonate kinase](https://blast.ncbi.nlm.nih.gov/Blast.cgi#alnHdr_808376609) | POR33036 | *Tolypocladium paradoxum* | phosphomevalonate kinase (ERG 8) |
| Sterol | 10 | 700913-702433 | 0 | 95 | 63 | Squalene epoxidase | CRK44748 | *Verticillium longisporum* | Squalene epoxidase (ERG 1) |
|  | 0 | 221818-223425 | 9e-151 | 99 | 48 | Hypothetical | XP_016640119. | *Scedosporium apiospermum* | 3-keto sterol reductase (ERG 27) |
|  | 12 | 76949-77519 | 3e-67 | 97 | 63 | Ergosterol biosynthesis protein 28 | OLN97423 | *Colletotrichum fioiriani* | Ergosterol biosynthesis protein 28 (ERG 28) |
|  | 8 | 1297859-1299460 | 0.0 | 99 | 70 | Delta (14)-sterol reductase | XP_016642425 | *Scedosporium apiospermum* | C-14-sterol reductase (ERG 24) |
|  | 10 | 339053-340190 | 7e-173 | 100 | 75 | C-4 methyl sterol oxidase | XP_016640914 | *Scedosporium apiospermum* | C4 sterol methyl oxidase (ERG 25) |
|  | 18 | 2218316-2219496 | e-126 | 79 | 67 | C-5 sterol desaturase | [XM_018809891.1](https://www.ncbi.nlm.nih.gov/nucleotide/XM_018809891.1?report=genbank&log$=nucltop&blast_rank=1&RID=GUGGXKMA015) | *Trichoderma gamsii* | C5 sterol desaturase (ERG 3) |
|  | 10 | 92795-93564 | 2e-113 | 100 | 67 | c-8 sterol isomerase | XP_003649780 | *Thielavia terrestris* | C8 sterol isomerase (ERG 2) |
|  | 45 | 467415-469235 | 0.0 | 86 | 76 | Delta (24(24(1)))-sterol reductase | [XM_009651622.1](https://www.ncbi.nlm.nih.gov/nucleotide/XM_009651622.1?report=genbank&log$=nucltop&blast_rank=1&RID=GUGGXKMA015) | *Verticillium dahliae* | C24 sterol reductase (ERG 4) |
|  | 17 | 162433-165817 | 0.0 | 99 | 67 | Cytochrome P-450 | KUI53848 | *Valsa mali* | Cytochrome P-450 (ERG 5) |
|  | 8 | 7859-9460 | 0.0 | 91 | 76 | Cytochrome P450 | [XM_018317745.1](https://www.ncbi.nlm.nih.gov/nucleotide/XM_018317745.1?report=genbank&log$=nucltop&blast_rank=1&RID=GUGGXKMA015) | *Purpureocillium lilacinum* | C24 sterol reductase (ERG 24) |
|  | 32 | 14961-16251 | 0.0 | 99 | 82 | C24 sterol methyltransferase | ELA25057 | *Colletotrichum gloeosporioides* | C24 sterol methyltransferase (ERG 6) |
|  | 39 | 206020-208370 | 0.0 | 99 | 72 | Lanosterol synthase | PKS07236 | *Lomentospora prolificans* | Lanosterol synthase (ERG 7) |
|  | 12 | 229698-231305 | 0.0 | 98 | 64 | Squalene synthase | XP_011395224 | Neurospora crassa | Squalene synthase (ERG 9) |
|  | 32 | 85513-89100 | 0.0 | 86 | 73 | 14-alpha-lanosterol demethylase | PKS10573 | *Lomentospora prolificans* | C14 demethylase (ERG 11) |
