# Supplementary material for: Characterization of the Ergosterol Biosynthesis Pathway in Ceratocystidaceae
Source: J Fungi (Basel). 2021 Mar 22;7(3):237. doi: 10.3390/jof7030237 (PMC8004197; doi:10.3390/jof7030237)
Supplement: Supplementary file 1 [file jof-07-00237-s001.zip › Supplementary file S-4.docx]

**Supplementary Table S-4**. Isolates numbers and genome sequence information for the species used for the sterol detection and quantification studies.

| **Species** | **Isolate number ^a^** | **GenBank Accession number** | **References** |
| --- | --- | --- | --- |
| *B. fagacearum* | CMW2656 | MKGJ00000000 | Wingfield *et al* 2016b |
| *C. adiposa* | CMW2573 | LXGU00000000 | Wingfield *et al* 2016a |
| *H. moniliformis* | CMW10134 | JMSH00000000 | Van der Nest *et al* 2014 |
| *T. punctulata* | BPI 893173 | LAEV00000000 | Wingfield *et al* 2015a |
| *D. virescens* | CMW17339 | LJZU000000000 | Wingfield *et al* 2015b |
| *E. polonica* | CMW20930 | LXKZ00000000 | Wingfield *et al* 2016a |
| *C. manginecans* | CMW17570 | JJRZ00000000 | Van der Nest *et al* 2014b |

^a^Isolates with CMW numbers may be obtained from the culture collection of the Tree Protection Cooperative Programme (TPCP), Forestry and Agricultural Biotechnology Institute (FABI), University of Pretoria, Pretoria, South Africa. Those with CBS and BPI numbers may be obtained from Centraalbureau voor Schimmel cultures, CBS Fungal Biodiversity Centre and the US National Fungus Collections, Systematic Botany and Mycology Laboratory, Maryland, U.S.A.
