# Supplementary material for: Characterization of the Ergosterol Biosynthesis Pathway in Ceratocystidaceae
Source: J Fungi (Basel). 2021 Mar 22;7(3):237. doi: 10.3390/jof7030237 (PMC8004197; doi:10.3390/jof7030237)
Supplement: Supplementary file 1 [file jof-07-00237-s001.zip › Supplementary file S-5.docx]

**Table 1**. Isolates used for the sensitivity test in this study.

| **Species** | **Isolate number ^a^** | **Fungicide used** | **Media** |
| --- | --- | --- | --- |
| *B. fagacearum* | CMW2656 | Triazole | MEA |
| *C. adiposa* | CMW2573 | Triazole | MEA |
| *Be. basicola* | CMW49352 | Triazole | MEA |
| *H. moniliformis* | CMW10134 | Triazole | MEA |
| *T. punctulata* | BPI 893173 | Triazole | MEA |
| *D. virescens* | CMW17339 | Triazole | MEA |
| *D. neocaledoniae* | CMW225392 | Triazole | MEA |
| *D. australis* | CMW2333 | Triazole | MEA |
| *E. polonica* | CMW20930 | Triazole | MEA |
| *A. xylebori* | CBS110.61 | Triazole | MEA |
| *Be. basicola* | CMW49352 | Triazole | MEA |
| *C. manginecans* | CMW17570 | Triazole | MEA |
| *C. fimbriata* | CMW 15049 | Triazole | MEA |
| *C. eucalypticola* | CMW 11536 | Triazole | MEA |
| *C. harringtonii* | CMW 14789 | Triazole | MEA |
| *C. smalleyii* | CMW 14800 | Triazole | MEA |
| *C. albifundus* | CMW 13980 | Triazole | MEA |
| *E. laricicola* | CMW 20928 | Triazole | MEA |
| *H. decipiens* | CMW 30855 | Triazole | MEA |
| *H. bhutanensis* | CMW 8217 | Triazole | MEA |
| *H. omanensis* | CMW 11056 | Triazole | MEA |
| *H. savannae* | CMW 17300 | Triazole | MEA |
| *T. musarum* | CMW 1546 | Triazole | MEA |

^a^Isolates with CMW numbers may be obtained from the culture collection of the Tree Protection Cooperative Programme (TPCP), Forestry and Agricultural Biotechnology Institute (FABI), University of Pretoria, Pretoria, South Africa. Those with CBS and BPI numbers may be obtained from Centraalbureau voor Schimmel cultures, CBS Fungal Biodiversity Centre and the US National Fungus Collections, Systematic Botany and Mycology Laboratory, Maryland, U.S.A.
